# The role of brain structure in the association between pubertal timing and depression risk in an early adolescent sample (the ABCD Study®): A registered report

**DOI:** 10.1016/j.dcn.2023.101223

**Published:** 2023-02-24

**Authors:** Niamh MacSweeney, Judith Allardyce, Amelia Edmondson-Stait, Xueyi Shen, Hannah Casey, Stella W.Y. Chan, Breda Cullen, Rebecca M. Reynolds, Sophia Frangou, Alex S.F. Kwong, Stephen M. Lawrie, Liana Romaniuk, Heather C. Whalley

**Affiliations:** a Division of Psychiatry, University of Edinburgh, Edinburgh, United Kingdom; b School of Psychology and Clinical Language Sciences, University of Reading, United Kingdom; c Institute of Health and Wellbeing, University of Glasgow, Glasgow, United Kingdom; d University of Edinburgh/British Heart Foundation Centre for Cardiovascular Science, Queen’s Medical Research Institute, Edinburgh, United Kingdom; e Department of Psychiatry, Icahn School of Medicine at Mount Sinai, New York, NY, USA; f Department of Psychiatry, Djavad Mowafaghian Centre for Brain Health, University of British Columbia, Vancouver, BC, Canada; g MRC Integrative Epidemiology Unit at the University of Bristol, United Kingdom; h Population Health Sciences, Bristol Medical School, University of Bristol, United Kingdom

**Keywords:** Pubertal timing, Adolescent depression, Brain structure, ABCD Study

## Abstract

**Background:**

Earlier pubertal timing is associated with higher rates of depressive disorders in adolescence. Neuroimaging studies report brain structural associations with both pubertal timing and depression. However, whether brain structure mediates the relationship between pubertal timing and depression remains unclear.

**Methods:**

The current registered report examined associations between pubertal timing (indexed via perceived pubertal development), brain structure (cortical and subcortical metrics, and white matter microstructure) and depressive symptoms in a large sample (N = ∼5000) of adolescents (aged 9–13 years) from the Adolescent Brain Cognitive Development (ABCD) Study. We used three waves of follow-up data when the youth were aged 10–11 years, 11–12 years, and 12–13 years, respectively. We used generalised linear-mixed models (H1) and structural equation modelling (H2 & H3) to test our hypotheses.

**Hypotheses:**

We hypothesised that earlier pubertal timing at Year 1 would be associated with increased depressive symptoms at Year 3 (H1), and that this relationship would be mediated by global (H2a-b) and regional (H3a-g) brain structural measures at Year 2. Global measures included reduced cortical volume, thickness, surface area and sulcal depth. Regional measures included reduced cortical thickness and volume in temporal and fronto-parietal areas, increased cortical volume in the ventral diencephalon, increased sulcal depth in the pars orbitalis, and reduced fractional anisotropy in the cortico-striatal tract and corpus callosum. These regions of interest were informed by our pilot analyses using baseline ABCD data when the youth were aged 9–10 years.

**Results:**

Earlier pubertal timing was associated with increased depressive symptoms two years later. The magnitude of effect was stronger in female youth and the association remained significant when controlling for parental depression, family income, and BMI in females but not in male youth. Our hypothesised brain structural measures did not however mediate the association between earlier pubertal timing and later depressive symptoms.

**Conclusion:**

The present results demonstrate that youth, particularly females, who begin puberty ahead of their peers are at an increased risk for adolescent-onset depression. Future work should explore additional biological and socio-environmental factors that may affect this association so that we can identify targets for intervention to help these at-risk youth.

## 1 Introduction

Adolescence is a period of increased vulnerability to mental health conditions, particularly internalising difficulties such as depression (Malhi and Mann, 2018, Thapar et al., 2012). Earlier-onset of depression is associated with a more severe illness course (Thapar et al., 2012) and with a range of psychosocial and physical difficulties which perpetuate across the lifespan (Clayborne et al., 2019, Fergusson and Woodward, 2002, Naicker et al., 2013). Given the emergence of depression during the adolescent period, the role that pubertal development may play in this heightened vulnerability has garnered increasing attention (Graber, 2013, Hamlat et al., 2019, Pfeifer and Allen, 2021, Ullsperger and Nikolas, 2017). Earlier pubertal timing has been associated with increased risk for depression in both males and females (Graber, 2013, Hamlat et al., 2020, Mendle et al., 2010, Ullsperger and Nikolas, 2017). Further, genetic studies have found that earlier age of menarche is implicated in depression (Howard et al., 2019). Adolescence is also a time of immense neurobiological change (Mills et al., 2016, Tamnes et al., 2017, Vijayakumar et al., 2016) and brain structural differences have been found in both adults (Schmaal et al., 2017, Schmaal et al., 2020, Shen et al., 2017, Shen et al., 2019) and adolescents (Schmaal et al., 2017, Shen et al., 2021) with depression. However, the role that neural mechanisms may play in the relationship between pubertal timing and depression risk is not well understood. Here, we therefore examine whether brain structure mediates the association between pubertal timing and depressive symptoms in a large sample of adolescents (aged 9–13 years) from the Adolescent Brain Cognitive Development (ABCD) Study®.

### 1.1 Defining and measuring pubertal timing

Pubertal timing measures pubertal development relative to same-age, same-sex peers, such that an individual can be categorised as developing ahead (early), in-line (on-time) or after (late) their peers. Measures of pubertal timing are most often derived from methods used to assess pubertal status, such as the Pubertal Development Scale (PDS; Petersen et al., 1988) and Tanner Stage Line Drawings (TS; Marshall and Tanner, 1969, Marshall and Tanner, 1970). However, other measures used include age of menarche and sex hormone measures (Goddings et al., 2019, Ullsperger and Nikolas, 2017). Pubertal maturation as assessed by the PDS and TS focuses on the development of secondary sex characteristics (e.g., testicular, breast, and pubic hair development), which stem directly from changes in sex hormones. These measures are completed by a clinician (TS), or via self- (or parent-) report (PDS/TS). Most often, a pubertal timing score is derived by regressing a pubertal status score on chronological age to calculate a sex-specific residual for each person (Barendse et al., 2021, Dorn and Biro, 2011, Mendle et al., 2010, Ullsperger and Nikolas, 2017). The residual score represents how much an individual’s pubertal development deviates from what is expected for their age with positive and negative scores indicating earlier and later timing, respectively. It is worth noting that pubertal development consists of two phases: adrenarche, usually occurring between the ages 6–9 years (Biro et al., 2014), and gonadarche, which typically takes place between the ages 9–14 years for females and 10–15 years for males.

### 1.2 Pubertal timing and psychopathology

Historically, research on pubertal timing effects on psychopathology has highlighted that youth, particularly females (Graber, 2013, Hamlat et al., 2019, Hamilton et al., 2014), who undergo puberty earlier than their peers are at an increased risk for psychopathology (Conley et al., 2012, Ge and Natsuaki, 2009, Hamilton et al., 2014). However, a recent meta-analysis suggests that earlier pubertal timing is detrimental to both sexes and that later pubertal timing is not significantly associated with psychopathology (Ullsperger and Nikolas, 2017). Although a number of conceptual models (Brooks-Gunn et al., 1985, Brooks-Gunn et al., 1994, Petersen et al., 1988) have been proposed to explain the association between earlier pubertal timing and increased risk for psychopathology, the “maturation disparity hypothesis” (Brooks-Gunn et al., 1985, Ge et al., 2001, Ge and Natsuaki, 2009), has received the most empirical support (Graber, 2013, Ullsperger and Nikolas, 2017). The maturation disparity hypothesis posits that early developing youth experience psychological distress due to an incongruity between their accelerated physical development and asynchronous maturation of cognitive and emotional brain regions.

Importantly, the psychological and social changes that occur during adolescence such as heightened self-awareness and social sensitivity (Blakemore and Mills, 2014, Pfeifer and Peake, 2012), increased risk-taking behaviour and impulsivity (Bjork and Pardini, 2015, Defoe et al., 2015, Romer, 2010) as well as greater peer influence on behaviour (Albert et al., 2013, Blakemore, 2018, Knoll et al., 2015) are likely underpinned by the distinct developmental trajectories of temporal and limbic areas (involved in emotion and reward processing) and prefrontal regions (involved in cognitive control) (Albert et al., 2013, Casey et al., 2008, Mills et al., 2014, Steinberg, 2008). It has been postulated that earlier developing youth therefore experience a greater discordance in the mismatch between the earlier developing affective regions and the more protracted development of cognitive regions (Ge and Natsuaki, 2009, Ullsperger and Nikolas, 2017), which may place them at an increased risk for mental health difficulties. Given that the onset of puberty is about 18 months earlier for females than males, this maturation disparity hypothesis may also explain the preponderance of depression (2:1) in females compared to males from adolescence onwards (Conley et al., 2012, Hankin, 2006, Hankin, 2015, Hankin and Abramson, 1999). Although the maturation disparity hypothesis best accounts for the extant findings, a more nuanced model that considers the role of biological and psychosocial factors as potential mediators or moderators in the association between earlier pubertal timing and increased risk for psychopathology is needed.

### 1.3 Pubertal timing and brain structure

Research on typical neurodevelopment demonstrates a reduction in grey matter volume and cortical thickness during adolescence, while cortical surface area increases throughout childhood before plateauing by mid-adolescence, and slightly decreasing thereafter (Bethlehem et al., 2022, Ducharme et al., 2016, Mills et al., 2016, Vijayakumar et al., 2016, Wierenga et al., 2014). These patterns of human brain development, were recently evidenced in a collaborative paper involving > 100 studies and > 123,000 MRI scans (Bethlehem et al., 2022), which is the largest aggregated sample to date. However, research has also shown that pubertal development impacts neurodevelopment beyond age-related changes (Vijayakumar et al., 2018). For example, a number of studies demonstrate extensive negative associations between pubertal timing (indexed via physical and hormonal measures) and cortical volume and thickness, mainly in regions implicated in cognitive control, decision making, and emotion regulation, such as the prefrontal cortex, anterior cingulate cortex, and the temporal lobe (Koolschijn et al., 2014, Pfefferbaum et al., 2016). Notably, few studies have examined surface area changes during puberty as surface area is often obscured when investigating volumetric estimates — a product of cortical surface area and thickness (Vijayakumar et al., 2016). Given that surface area and cortical thickness reflect distinct neurobiological processes (Wierenga et al., 2014) and are genetically independent of each other (Winkler et al., 2010), examining pubertal timing in relation to surface area maturation may reveal novel associations.

Regarding associations between subcortical measures and pubertal development, research has focused on the amygdala, hippocampus, and striatal regions given their role in emotion regulation, reward processing, and decision making (Bhanji and Delgado, 2014, Dalgleish, 2004). A number of cross-sectional and some longitudinal studies have reported that more advanced pubertal maturation is associated with an increase in volume of the amygdala and hippocampus and a decrease in volume in striatal areas (Blanton et al., 2012, Hu et al., 2013, Goddings et al., 2014, Goddings et al., 2019). Although these findings provide insight into the role of puberty in subcortical brain development, there is a dearth of research that specifically examines pubertal timing (i.e., pubertal development relative to same-age, same-sex peers) (Goddings et al., 2019) and its association with brain morphological changes (Koolschijn et al., 2014, Neufang et al., 2009, Peper et al., 2009). Further, longitudinal data has shown that there are unique but co-existing age effects that complicate examining the relationship between puberty and structural brain development (Goddings et al., 2019). For example, a recent longitudinal study demonstrated a positive linear association between perceived pubertal maturation (indexed via TS) and the hippocampus, amygdala, caudate and pallidum. However, these associations did not remain significant when age was controlled for (Vijayakumar et al., 2021). Further inconsistencies have emerged in the literature when utilising different measures of pubertal development (Koolschijn et al., 2014, Vijayakumar et al., 2018), and also in studies using large age ranges (Satterthwaite et al., 2014, Urošević et al., 2014). There is also some research suggesting that pituitary gland volume mediates the association between earlier pubertal timing and increased risk for depression in adolescence but more research is needed on this topic (Whittle et al., 2012). Additionally, the current literature does not allow for the identification of clear sex differences in the association between cortical and subcortical structure and pubertal timing, likely owing to the paucity of longitudinal, large-scale research in this area (Herting and Sowell, 2017, Vijayakumar et al., 2018).

There is less research examining the association between pubertal development and white matter microstructure (Goddings et al., 2019) and findings are mixed (Vijayakumar et al., 2018). There is some degree of support for a positive association between pubertal timing and fractional anisotropy (FA; Herting et al., 2012; Peper et al., 2015). However, findings have been inconsistent when considering the relation between pubertal hormones and FA, and between all indices of pubertal development (physical maturation and hormonal measures) and mean diffusivity (MD; Herting et al., 2012; Peper et al., 2009). These discrepancies may be attributed to the various diffusion tensor imaging (DTI) techniques employed and the relatively small sample sizes. Future large-scale neuroimaging research that leverages harmonised protocols and considers the unique and contemporaneous effect of age is needed to disentangle the associations between pubertal timing and white matter microstructure.

Large, population-based research projects, such as the ABCD Study®, directly address limitations of earlier research (e.g., small sample sizes, inconsistent protocols) and will allow us to investigate how brain changes across adolescence are related to developmental outcomes, especially the emergence of mental health difficulties (Casey et al., 2018). The ABCD Study includes magnetic resonance imaging (MRI) data, assessments of psychiatric disorders, and hormonal and physical puberty measures in 9–10-year-old US children at baseline (N = ∼11,800). Our previous work with the ABCD Study® explored the temporal origins of depression-related imaging features and demonstrated that depression ratings in early adolescence were associated with similar cortical and white matter microstructural differences to those seen in adult samples (Shen et al., 2021). These findings suggest that neuroanatomical abnormalities may be present early in the disease course. However, the cascade of neurobiological changes associated with the onset of puberty may have an important role in risk for depression and may allow further mechanistic insight into the origins of these depression-related imaging features (Dahl et al., 2018, Pfeifer and Allen, 2021).

### 1.4 Current study

While research has shown that earlier pubertal timing is associated with an increased risk for depression, the underlying neurobiological mechanisms remain unclear. The goal of the present study was to investigate whether brain structure (cortical and subcortical metrics, and white matter microstructural measures) mediates the association between earlier pubertal timing (indexed via perceived physical development) and increased depressive symptoms in a young adolescent sample. We first tested if earlier pubertal timing when youth were aged 10–11 years (Year 1) was associated with higher depressive symptoms two years later when they were aged 12–13 years (Year 3). We then examined whether specific brain structural metrics at Year 2 (identified via our pilot analyses, see Supplementary Information), mediated the association between earlier pubertal timing and later depressive symptom severity. Given the differences in the average age of puberty-onset for males and females, we ran our models separately for males and females.

Specifically, our key hypotheses were that earlier pubertal timing at Year 1 would be associated with increased depressive symptoms at Year 3 (**H1**), and that this relationship would be mediated by global (H2 a-b) and regional measures **(H3a-g)** outlined in Table 1. These regions of interest were consistent with existing literature on puberty- and depression-related imaging features in adolescence (Schmaal et al., 2017, Shen et al., 2021, Vijayakumar et al., 2018). We did not make formal hypotheses about sex differences in the current study due to inconsistent findings in the literature.

**Table 1:** Hypotheses tested in this registered report.

Research Q	Hypothesis	Analysis Test	Effect of Interest	Threshold for evidence
Is earlier pubertal timing associated with later depression?	H1: Earlier pubertal timing will be associated with later higher depressive symptoms	Generalised linear mixed effects model	Beta value and p value	ß ≥ 0.01 and p ≤ 0.05
Does brain structure mediate the association between earlier pubertal timing and later depression?	Informed by our pilot analyses, the association between earlier pubertal timing and increased depressive symptoms will be mediated by: *Global measures* **H2a**: Reduced global cortical volume, surface area, thickness and sulcal depth **H2b:** Reduced global FA *Regional measures* **H3a:** Reduced cortical thickness in temporal regions, namely, the middle temporal gyrus and insula **H3b:** Reduced cortical thickness in frontal regions namely, the lateral orbito-frontal cortex and middle frontal gyri **H3c:** Reduced cortical volume in temporal regions, namely, middle temporal gyrus and bank of the superior temporal sulcus **H3d:** Reduced cortical volume in fronto-parietal regions, namely, the middle frontal and postcentral gyri **H3e** Reduced FA in the cortico-striatal tract and corpus collosum **H3f:** Increased sulcal depth in the pars orbitalis **H3g:** Increased volume in the ventral diencephalon	Multi-level structural equation model	Indirect effect in mediation model	ß ≥ 0.01 and p ≤ 0.05

The results of this multi-modal study will inform our understanding of how pubertal timing and brain structure may be associated with depression during adolescence. Undertaking this project as a registered report with shared analytic code applied to an openly available dataset will further increase the replicability and reproducibility of this work.

## 2 Materials and methods

### 2.1 Participants

The data used in the current study were drawn from the ABCD curated annual data release 4.0. and used Year 1, Year 2, and Year 3 follow up data. ABCD participants were recruited from 21 sites across the United States (Garavan et al., 2018). Approximately N = ∼11,800 9–10-year-olds participated in the baseline assessment. We included individuals with quality-controlled pubertal development measures (physical) at Year 1 and quality-controlled brain imaging measures (cortical and subcortical size/metrics, and white matter measures) at Year 2. Missing depression outcome and covariate data were handled using appropriate methods (see *Missing Data* section). This resulted in a final sample of approximately N = ∼5000 individuals, which represents about 50% of the full sample. This smaller sample size can be attributed to the partial follow-up data available at the time of the 4.0 data release (Fall 2021).

To inform hypotheses for the current registered report, specifically the brain regions of interest (ROIs), we conducted pilot analyses using data from the baseline timepoint, when youth were aged 9–10 years (N = 9339, males = 4802, females = 4537) from data release 4.0. Participants were included in the pilot analyses if they had quality controlled pubertal development, depression, and brain imaging measures. Given that the main analyses did not use any baseline data, we did not anticipate that this decision would significantly impact our findings. Findings from the pilot analyses are reported in the Supplementary Information.

### 2.2 Measures

All variables (excluding imaging variables) as per the NDA ABCD data dictionary/Data Exploration and Analysis Portal (DEAP) portal field names can be found in Table 2 below.

**Table 2:** Name and description of study variables used in this registered report.

Field name (s)	Description
pds_1_p; pds_2_p; pds_3_p; pds_f4_p; pds_f5b_p	Caregiver: PDS female items, which were summed to generate PDS total score.
pds_1_p; pds_2_p; pds_3_p; pds_m4_p; pds_m5_p.	Caregiver: PDS male items, which were summed to generate PDS total score
cbcl_scr_syn_withdep_r	CBCL withdrawn-depressed syndrome subscale raw score
age_years	Age of child in years
anthro_bmi_calc	Body mass index (BMI) (DEAP field name, non-NDA)
race.6level	6-level derived race variable (white, black, Asian, AIAN/NHPI, other, mixed)
demo_comb_income_p	Household income
asr_scr_depress_r	Parental mood
acs_raked_propensity_score	Imputed raked propensity weight. The raked propensity weight merges the ACS and ABCD data (with missing data imputed), estimates the propensity model, computes and scales/trims the propensity weights and finally rakes the scaled weights to final ACS control totals by age, sex and race/ethnicity.
site_id_l	ABCD study site
mri_info_deviceserialnumber	Scanner ID
dmri_dti_meanmotion	DTI average framewise displacement in mm
rel_family_id	Family ID

Field names are the column names in the original curated ABCD data release or in the DEAP portal.

#### 2.2.1 Independent variable — pubertal timing measure

Protocols previously outlined by Cheng et al. (2021) and Herting et al. (2021) were consulted in the preparation of the pubertal timing measures.

The Pubertal Development Scale (PDS) was used to examine the perceived development of secondary sex characteristics such as growth spurts, body hair growth, skin changes, breast development and menarche in girls, and voice changes and growth of facial hair in boys. In line with existing work on puberty measures in the ABCD Study, and given previous research showing that youth tend to over-report their perceived physical development at younger ages (Schlossberger et al., 1992), caregiver PDS report was utilised instead of child self-report in the current analysis. The PDS includes five-items, and each characteristic is rated on a 4-point scale (1 = no development; 2 = development has barely begun; 3 = development is definitely underway; and 4 = development is complete; except menstruation, which is coded 1 = has not begun, 4 = has begun). Thus, higher scores reflect more advanced pubertal maturation. We did not examine age of menarche in the current analysis due to the small number of females (3% and 13%) in the ABCD sample that have reached this developmental milestone at baseline and Year 1 data collection, respectively (Herting et al., 2021).

In line with existing research on pubertal timing, the PDS total score was regressed on age for girls and boys separately and the standardised residual obtained constituted the continuous measure of pubertal timing (Dorn et al., 2006, Hamilton et al., 2014). Only participants with complete data for 5/5 PDS items were included in the analysis.

**Fig. 1:**
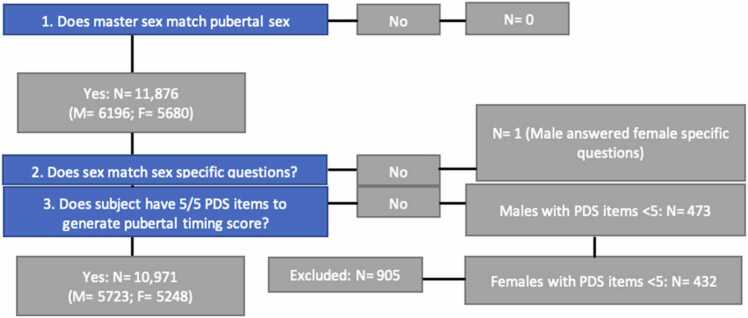
Pubertal Development Scale quality checking decision tree at Year 1. (curated annual release 4.0). Notes: Master sex = Q: Biological sex of subject, Answer: Male, Female, Other, Not Reported.

Changes to the sample size at each stage of the quality control process can be found in Fig. 1.

#### 2.2.2 Dependent variable — depressive symptoms

Depressive symptoms (DS) for children were assessed using the Child Behaviour Check List (CBCL) parent report. The CBCL is one of the most widely used measures to examine internalising and externalising difficulties in youth (Achenbach and Rescorla, 2004). It comprises raw scores as well as standardised (T-scores) based on national norms in young people aged 6–18 years. We quantified current depressive symptoms using the CBCL “withdrawn-depressed” syndrome subscale raw scores, which examine depressive symptoms within the past two weeks. Raw scores were chosen over T-scores for our analyses because they reflect all the variation in symptoms that occur in the sample. Due to the substantial percentage of individuals in a normative sample who obtain low scores on the CBCL syndrome subscales, the T-score assignments begin at 50 which means that all individuals in the lowest 50% are assigned a T-score of 50.

#### 2.2.3 Hypothesised mediator — brain structural measures

Brain imaging data were acquired and processed by the ABCD team. A 3 T Siemens Prisma, General Electric 750 or Phillips scanner was used for data acquisition. A unified protocol for the scanning was used to harmonise between sites and scanners (Casey et al., 2018). Protocols used for data acquisition and processing are described elsewhere (Casey et al., 2018, Hagler et al., 2019). In brief, T1-weighted data was acquired by magnetisation-prepared rapid acquisition gradient echo scans with a resolution of 1 × 1 × 1 mm^3^, which was used for generating cortical and subcortical structural measures, and diffusion-weighted data was obtained by high angular resolution diffusion tensor imaging (DTI) scans, used for generating white matter microstructural measures.

Imaging data was quality controlled according to recommended QC criteria outlined by ABCD in the 4.0 release notes: “MRI Quality Control (QC) and Recommended Image Inclusion Criteria”. ABCD have created a data structure *abcd_imgincl01* that provides modality-specific summary imaging inclusion flags that indicate whether an individual meets all QC criteria for the modality, and are scored as 1 = include, 0 = exclude. These summary variables account for factors such as imaging QC and post-processing (see public release notes for full description). Public release notes are available here. We included individuals that met all the recommended inclusion criteria (i.e., score = 1) on the “imgincl_t1w_include” variable for the T1w data and the “imgincl_dmri_include” variable for the DTI data. To account for additional motion artefact in the DTI data, we included a measure of mean framewise displacement (variable name: dmri_dti_meanmotion) in our DTI models.

Three types of brain structural measures were used in the present study: grey matter cortical and subcortical metrics, and white matter microstructural measures. The derivation of brain structural measures followed a hierarchical order from global measures at the whole-brain level to individual structures.

Cortical measures were generated using Freesurfer 5.3.0 (https://surfer.nmr.mgh.harvard.edu/fswiki/FreeSurferWiki). Four types of cortical measures were used: surface area, thickness, volume and sulcal depth. First, global measures were generated for each cortical measure over the whole brain. The Desikan-Killiany atlas was used for parcellation of 34 bilateral cortical structures and 16 bilateral subcortical structures. For bilateral brain structures, we generated an average measure across the left and right hemisphere to use in our analyses.

White matter microstructural measures included fractional anisotropy (FA) and mean diffusivity (MD). Global measures of FA and MD were generated over the whole brain. The AtlasTrack was used to map boundaries of the 14 bilateral and 3 unilateral major tracts (Hagler et al., 2009). FA/MD values were then derived for each tract.

### 2.3 Covariates

Research examining how puberty is related to developmental processes and outcomes indicates that several factors may shape these associations. Examining variability in these constructs is crucial to understanding their unique contributions to developmental and psychological outcomes (Saragosa-Harris et al., 2022). For example, differences in race/ethnicity and body mass index (BMI) have been associated with early pubertal timing (e.g., earlier age of menarche and onset of breast development) (Biro et al., 2013, Chumlea et al., 2003) and an increased risk for depression (Anderson and Mayes, 2010, Quek et al., 2017). Further, youth raised in families with low socioeconomic status, especially those with significant financial hardship, are at an increased risk for psychopathology (Bradley and Corwyn, 2002, Herting et al., 2021, Peverill et al., 2021). Research has also demonstrated that parental mood can influence the reporting of their child’s psychopathology (Maoz et al., 2014).

Although the imaging QC protocol outlined by ABCD excluded participants with excessive head motion across all imaging modalities (i.e., structural and DTI), motion-related confounds have been found to systematically impact structural connectivity measures derived from DTI data (Baum et al., 2018). Children of the same age may exhibit developmental differences in cranial or brain size, which need be considered to determine whether regional differences are independent of global effects (Mills et al., 2016, O’Brien et al., 2011). While there is currently no consensus on whether to use intracranial volume (ICV) or whole brain volume (WBV), some research suggests that WBV may be a more viable measure to use as it has been found to be more stable across developmental samples than ICV (Mills et al., 2016). The importance of controlling for site effects to account for inter-site variability has been well documented (Feaster et al., 2011). Although ABCD data collection took place at 21 sites, 30 different MRI scanners were used during data collection as some sites had more than one scanner. Therefore, the potential for variability across scanners is also important to consider (Saragosa-Harris et al., 2022). As ABCD has been oversampled for twins and siblings, it is important to account for relatedness between individuals when using the related sample (Gelman and Hill, 2006, Iacono et al., 2018).

Taking these findings together, when modelling the relationships between pubertal timing, brain structure and depressive symptoms, we included the following variables as *fixed effects* in our models: age, race/ethnicity, BMI, household income; parental current mood, and a DTI average framewise displacement measure (for DTI models only). We included family ID and site (for non-imaging models) and scanner ID (for all other models) as *random effects.*

### 2.4 Consideration of outcome neutral conditions

Effect sizes (β values) and FDR (false discovery rate) corrected p values (where appropriate) were the main parameters of interest in the main analyses of the current study. The minimum effect size of interest, β ≥ 0.01 and a p ≤ 0.05, was considered statistically significant. This was informed by our previous work using the ABCD sample, which examined baseline cross-sectional brain structural associations with depression ratings in adolescence (Shen et al., 2021).

## 3 Data analysis plan

### 3.1 Main analyses

All analyses were conducted in R Version 4.1. and Mplus Version 8.8. Scripts for all analyses can be found at https://github.com/niamhmacsweeney/ABCD_puberty_depression. Supplementary Data (including model outputs in R and Mplus) and the approved Stage 1 protocol for this registered report can be found on the OSF repository for this project here.

**Fig. 2:**
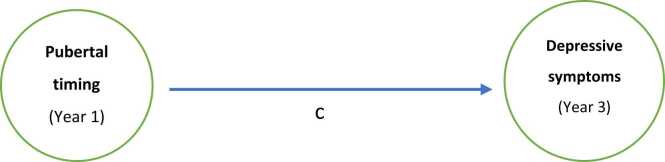
Effect of pubertal timing on depressive symptoms without considering mediation (c: total effect).

**Fig. 3:**
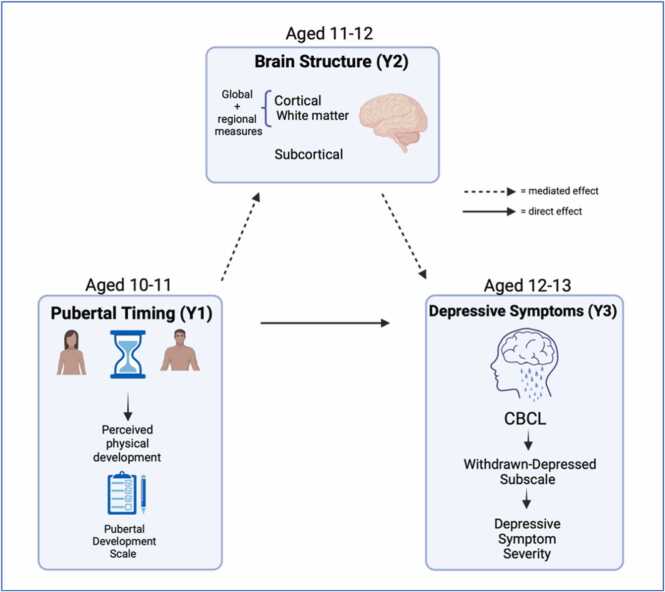
Effect of pubertal timing on depressive symptoms including mediation of brain structure.

The analytic approach comprised two steps: H1) examine the associations between pubertal timing and depressive symptoms in adolescents (see Fig. 2) and H2 and H3) determine whether brain structural measures (identified via pilot analyses) mediate this association (see Fig. 3). All hypotheses are outlined in Table 1.

We note that regardless of the effect sizes for our first association test (i.e., the total effect), we still conducted the mediation analysis in an attempt to accurately quantify any indirect effects (Agler and De Boeck, 2017). Further, although the models in our pilot analyses were run separately for males and females, and thus generated some varying ROIs, our main analyses used *all* the ROIs identified from both male and female models due to non-hypothesised sex differences in the current study.

When testing our hypotheses, we first ran our base model and if results met our specified threshold for evidence (see Table 1), we ran our full model structure. This allowed us to explore whether our main associations were attenuated by the presence of additional covariates.

Hypothesis 1 (H1): Pubertal timing → depressive symptoms.

Independent variable: Pubertal timing at Year 1 (youth aged 10–11 years). This was indexed by the PDS, which is a continuous measure.

Dependent variable: Youth current depressive symptoms at Year 3 (aged 12–13 years), as reported by caregiver. Depressive symptoms were indexed using the CBCL “withdrawn-depressed” syndrome subscale, which is in count data format.

Generalised linear mixed effects models (GLM) were conducted to test the associations, using the ‘lmerTest’ function in R (Kuznetsova et al., 2017). Models to test H1 are listed in Table 3.

**Table 3:** Model specifications for pubertal timing and depressive symptoms association.

Sex specific models	Covariates (Base model)	Covariates (Full model)
Depressive symptoms ∼ pubertal timing	Fixed: age, race/ethnicity Random: family, site	Fixed: age, race/ethnicity, BMI, site, household income, parental current mood Random: family, site


Models were conducted for males and females separately.

Total number of tests across all levels of model adjustment: **2** (females) and **2** (males).

Hypothesis 2 (H2): Pubertal timing → brain structural measures -> depressive symptoms.

We tested whether brain structural ROIs measured at Year 2 partially and significantly mediated associations between pubertal timing at Year 1 and depressive symptoms at Year 3 (see Fig. 3). ROIs were determined based on our pilot analyses, which are detailed in the Supplementary Information.

From our pilot analyses, several brain structural measures were found to be significantly associated with pubertal timing and depressive symptoms, and thus informed the following hypotheses:

The association between earlier pubertal timing and increased depressive symptoms would be mediated by:

### Global measures

• H2a: Reduced global cortical volume, surface area, thickness and sulcal depth.
• H2b: Reduced global FA

### Regional measures

• H3a: Reduced cortical thickness in temporal regions, namely, the middle temporal gyrus and insula.
• H3b: Reduced cortical thickness in frontal regions namely, the lateral orbito-frontal cortex and middle frontal gyrus.
• H3c: Reduced cortical volume in temporal regions, namely, middle temporal gyrus and bank of the superior temporal sulcus.
• H3d: Reduced cortical volume in fronto-parietal regions, namely, the middle frontal and postcentral gyri.
• **H3e** Reduced FA in the cortico-striatal tract and corpus collosum.
• **H3f:** Increased sulcal depth in the pars orbitalis
• **H3g:** Increased cortical volume in the ventral diencephalon.

We ran mediation analysis using multi-level structural equation modelling (MLSEM) with Mplus software (Preacher et al., 2010) and via the “MplusAutomation” package in R (Hallquist and Wiley, 2018). MLSEM enables the stratification of within individual, between family and site/scanner variance, therefore allowing us to capture these random effects.

This model characterised associations between pubertal timing, brain structural ROIs and depressive symptoms. We undertook single and multiple mediator models, depending on the brain ROI. Multiple mediator models allowed us to examine the proportion of variance in the pubertal timing-depression associations uniquely explained by all brain structural ROIs and allowed for comparisons between different ROIs. For this analysis, we simultaneously entered individual brain structural ROIs as covarying mediators. A combined cluster variable was used to model random effects in Mplus due to model parameter requirements.

The primary outcomes of interest were the direct effect between the pubertal timing measure and depressive symptoms, and the indirect paths between these two variables that are mediated by brain structural ROIs. Statistical significance of this indirect effect was used to indicate that a significant mediation of the total effect is present. An effect was considered statistically significant when p ≤ 0.05 and there was a minimum effect size (β value ≥ 0.01). As outlined in the “consideration of outcome neutral conditions*”* section, this minimum effect size was based on our previous work where effect sizes for brain structural associations with depression ratings in the ABCD sample were found to be in the region of 0.01–0.03 (Shen et al., 2021). Bootstrapping with 1000 repetitions was used to calculate robust standard errors.

The Base model included age, race/ethnicity, and DTI motion as fixed effects, and family ID and scanner ID as random effects. The full model included the same random effects as the base model but with the additional fixed effects: WBV, BMI, household income, and parental current mood.

### 3.2 Sensitivity analyses

In addition to the main models, we conducted sensitivity analysis to examine the association between earlier pubertal timing and the potential change (or rather worsening) of depressive symptoms between timepoints (i.e., Year 1 and Year 3), by including Year 1 depressive symptoms as an additional covariate in our full model as a sensitivity analysis. Further, to examine potential demographic and socio-economic bias in the selection of ABCD participants, we included a population-weighting variable in our full model that calibrates ABCD weighted distributions to nationally representative controls from the American Community Survey (ACS).

### 3.3 Missing data

Missing outcome and covariate data were handled using appropriate methods (Matta et al., 2018). Multiple imputation by chained equations (MICE) was used to treat missing data for H1 using the “mice” package in R (Buuren and Groothuis-Oudshoorn, 2011). For H2 and H3, full information maximum likelihood (FIML) estimation in Mplus was used to handle missing data in our mediation analyses. As sensitivity analyses, we compared our imputed analysis approach to complete case analysis for H1.

### 3.4 Exploratory analyses

To identify any additional relevant brain structural measures that may not have been identified in the pilot analyses due to the use of baseline data only, we also undertook exploratory whole brain analysis to examine whether any other brain structural measures (at Year 2) mediated the association between pubertal timing (at Year 1) and depressive symptoms (at Year 3). Multiple comparison correction (FDR method) was applied using the “p.adjust” function in R and applied to each brain measure category separately. These analyses were considered post-hoc and thus reported as exploratory findings.

### 3.5 Project timeline

Our Stage One registered report obtained an in-principle acceptance in August 2022, and we submitted our Stage 2 manuscript for review in December 2022.

## 4 Results

Sample characteristics are presented in Table 4 with further information on the pilot analyses given in the Supplementary Information.

**Table 4:** Descriptive statistics for sample.

**Characteristic**	**F**, N = 2726^a^	**M**, N = 3001^a^	**p-value**^b^
**Age (Y1)**	10.98 (0.63)	11.00 (0.64)	0.082
**Age (Y2)**	11.96 (0.64)	11.98 (0.65)	0.2
* Missing (N)*	34	35	
**Age (Y3)**	12.87 (0.64)	12.91 (0.65)	0.021
**PDS total score**	10.37 (3.09)	7.72 (2.12)	< 0.001
**Youth depressive symptoms**	1.49 (2.19)	1.30 (1.90)	0.012
* Missing (N)*	46	46	
**Race/ethnicity**			0.072
* White*	1876 / 2696 (69.58%)	2175 / 2975 (73.11%)	
* Black*	296 / 2696 (10.98%)	278 / 2975 (9.34%)	
* Asian*	70 / 2696 (2.60%)	73 / 2975 (2.45%)	
* AIAN/NHPI*	23 / 2696 (0.85%)	17 / 2975 (0.57%)	
* Other*	105 / 2696 (3.89%)	111 / 2975 (3.73%)	
* Mixed*	326 / 2696 (12.09%)	321 / 2975 (10.79%)	
* Missing (N)*	30	26	
**BMI (Y1)**	19.43 (4.24)	19.43 (4.10)	0.8
* Missing (N)*	51	43	
**Household income**			0.6
*< $5000*	47 / 2558 (1.84%)	66 / 2813 (2.35%)	
* $5,000-$11,999*	63 / 2558 (2.46%)	69 / 2813 (2.45%)	
* $12,000-$15,999*	55 / 2558 (2.15%)	43 / 2813 (1.53%)	
* $16,000-$24,999*	89 / 2558 (3.48%)	111 / 2813 (3.95%)	
* $25,000-$34,999*	142 / 2558 (5.55%)	133 / 2813 (4.73%)	
* $35,000-$49,999*	212 / 2558 (8.29%)	233 / 2813 (8.28%)	
* $50,000-$74,999*	364 / 2558 (14.23%)	388 / 2813 (13.79%)	
* $75,000-$99,999*	404 / 2558 (15.79%)	438 / 2813 (15.57%)	
* $100,000-$199,999*	848 / 2558 (33.15%)	964 / 2813 (34.27%)	
*> $200,000*	334 / 2558 (13.06%)	368 / 2813 (13.08%)	
* Missing (N)*	168	188	
**DTI mean FD**	1.20 (0.43)	1.24 (0.53)	0.14
* Missing (N)*	566	476	
**Parent depressive symptoms**	3.96 (3.59)	3.95 (3.54)	> 0.9
* Missing (N)*	35	38	

Y1 = year 1; Y2 = year 2; Y3 = year 3. Youth depressive symptoms = CBCL withdrawn depressed total raw score; AIAN/NHPI = American Indian/Alaska Native/Native Hawaiian and other Pacific Islander; Household income = yearly gross household income; DTI mean FD = mean framewise displacement from year 2 DTI data; Parent depressive symptoms = Depressive Problems ASR DSM-5-Oriented Scale.

a Mean (SD); n / N (%)

b Wilcoxon rank sum test; Pearson's Chi-squared test

**Fig. 4:**
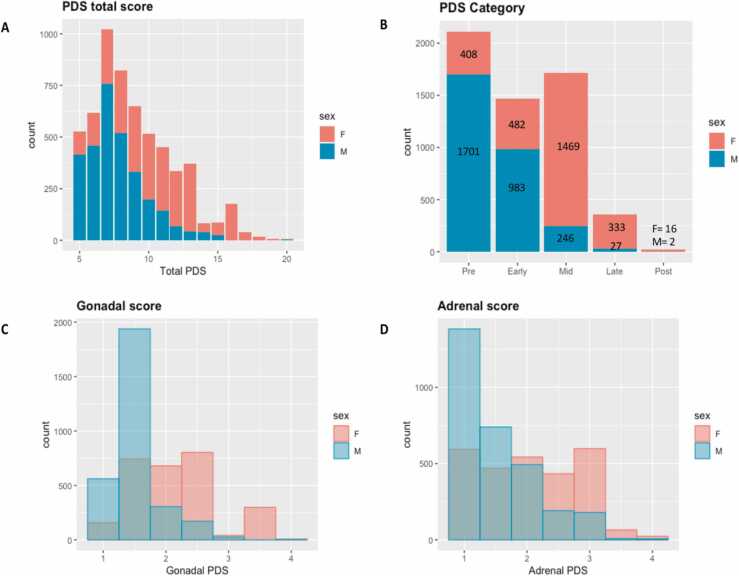
Frequencies (N) for parent summary scores from the Pubertal Development Scale (PDS). (A) Total pubertal development score; (B) PDS Category score counts ranging from pre- to post- pubertal. Note that PDS category score (variables: “pds_p_ss_female_category” and “pds_p_ss_male_category” data were not available for 60 participants in ABCD release 4.0 so N = 5667 (full sample N = 5727) for Figure 4.6b. (C) Gonadal score averaging gonadal PDS items and ranging from 1 = not begun to 4 = complete; (D) Adrenal score averaging adrenal PDS items ranging from 1 = not begun to 4 = complete.

Frequencies for perceived pubertal development and youth depressive symptoms, based on parent report, are shown in Fig. 4.

### 4.1 A note on the interpretation of effect sizes

All variables (except youth depressive symptoms [a count variable with a Poisson distribution] and the population weight propensity score) were converted to z-scores before entering our analyses to ensure they could be consistently interpreted across our hypotheses. The estimated ß coefficients should be interpreted as follows: for a unit change in the predictor variable, the difference in the logs of the expected counts is predicted to change by the respective ß value, while holding the other predictor variables in the model constant. To further aid the interpretation of our main results, we report incidence rate ratios (IRR) alongside the ß values and associated p-values. The incidence rate ratio is the exponentiated ß coefficient and can be interpreted as a relative risk.

### 4.2 Hypothesis 1

**Fig. 5:**
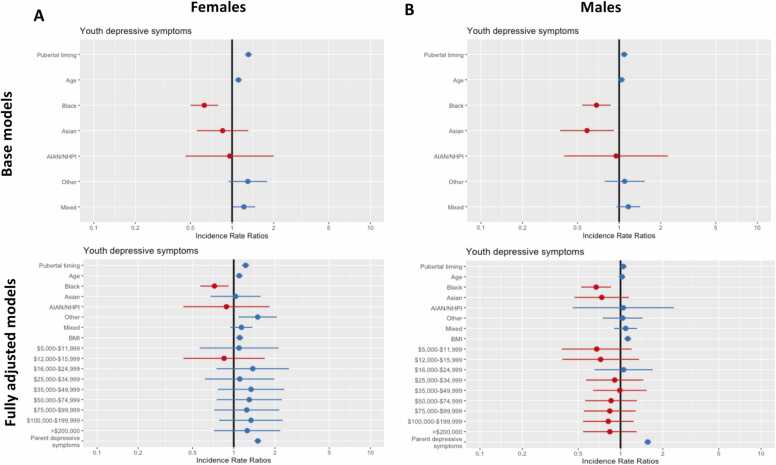
Hypothesis 1 results: Incidence Rate Ratios (IRRs) for the association between pubertal timing and youth depressive symptoms. Results for females are shown in (A) and males are shown in (B). Base models are shown in top panel and fully adjusted models are presented in the bottom panel. The neutral line or vertical intercept line is shown in bold and indicates no effect. Blue IRRs indicate a greater depression risk while red IRRs represent a decreased depression risk. Error bars represent 95% confidence interval.

Our first hypothesis tested whether earlier pubertal timing at year 1 (10–11 years) was associated with higher depressive symptoms at year 3 (12–13 years). In our base model, both females and males who started puberty earlier than their peers were more likely to report higher depressive symptoms two years later. Basic model: Females: ß = 0.27; [IRR = 1.31]; *p* < 2 × 10^-16^; males: ß = 0.08 [IRR = 1.09]; *p* = 0.005. In our fully adjusted model (with covariates BMI, household income, and parental depressive symptoms), our main effect size was attenuated for females but remained significant (ß = 0.20 [IRR = 1.22], *p* < 2 ×10^-16^). However, the observed effect size for males (ß = 0.04; [IRR = 1.045], *p* = 0.15) no longer met our threshold for evidence (see definition, Table 1). Thus, we find partial support for our first hypothesis. The results (reported using IRRs) for Hypothesis 1 are illustrated in Fig. 5. All statistics (e.g., ß values, IRRs, standard errors, and *p* values) for Hypothesis 1 are reported in Tables S1 & S2 in the Supplementary Information.

#### 4.2.1 Exploratory analyses related to Hypothesis 1

To explore whether there were specific aspects of pubertal development that were driving the association between earlier pubertal timing and increased depression risk, we ran two independent models (using H1 model set up) with adrenarcheal timing (AT) and gonadarcheal timing (GT) scores as predictors. For females, both AT and GT were significantly associated with later youth depression (Base model: AT: ß = 0.23 [IRR = 1.26]; *p* < 0.001; GT: ß = 0.24 [IRR = 1.28]; *p* < 0.001), and these effects remained significant in the fully adjusted model (AT: ß = 0.17 [IRR = 1.18]; *p* < 0.001; GT: ß = 0.17 [IRR = 1.19]; *p* < 0.001). For males, only AT was significantly associated with later youth depression (Base model: AT: ß = 0.10 [IRR = 1.11]; *p* = 0.001; GT: ß = 0.05 [IRR = 1.04]; *p* = 0.154). Neither association was significant in the fully adjusted model for males. The methods and results of this exploratory analysis are reported in full in the Supplementary Information (Tables S3 – S8).

### 4.3 Hypothesis 2 — global brain measures

Our second hypothesis tested whether global brain structural measures at Year 2 (specifically, lower global volume, cortical thickness, surface area, and sulcal depth (H2a) and FA (H2b)) mediated the association between earlier pubertal timing at Year 1 and higher depressive symptoms at Year 3. As reported in Table 5, for both females and males, we did not find support for these hypotheses in the current analyses due to the absence of an indirect effect (Females: H2a: ß = −0.001 [IRR = 0.99], *p* = 0.89; H2b: ß = 0.001 [IRR = 1.00], *p* = 0.57; Males: H2a: ß = 0.003 [IRR = 1.00], *p* = 0.43; H2b: ß = −0.001 [IRR = 0.99], *p* = 0.39) from our predictor (pubertal timing) to our outcome (youth depressive symptoms) through our hypothesised mediator (brain structure).

**Table 5:** Multiple mediator model results for Hypothesis 2 & 3. ROI = region of interest; PT = pubertal timing; DS = depressive symptoms.

				*Females*			*Males*		
Hypothesis	Mediator (ROI)	Type	Effect	Estimate	SE	p-value	Estimate	SE	p-value
H2a	Global cortical volume, thickness, surface area, sulcal depth	Indirect Direct Total	PT → ROI → DS PT → DS PT → DS	-0.001 0.223 0.22	0.007 0.032 0.031	0.898 < 0.001 < 0.001	0.003 0.080 0.083	0.004 0.03 0.029	0.425 0.008 0.004
H2b	Global fractional anisotropy	Indirect Direct Total	PT →ROI → DS PT → DS PT → DS	0.001 0.226 0.227	0.001 0.032 0.032	0.565 < 0.001 <0.001	-0.001 0.078 0.077	0.001 0.031 0.031	0.395 0.010 0.011
H3a	Cortical thickness of the middle temporal gyrus and insula	Indirect Direct Total	PT →ROI → DS PT → DS PT → DS	-0.008 0.231 0.223	0.005 0.032 0.031	0.059 < 0.001 < 0.001	-0.001 0.085 0.083	0.002 0.029 0.029	0.611 0.004 0.004
H3b	Cortical thickness of the lateral orbito-frontal cortex and middle frontal gyrus	Indirect Direct Total	PT → ROI → DS PT → DS PT → DS	-0.007 0.228 0.222	0.005 0.031 0.031	0.140 < 0.001 < 0.001	0.000 0.083 0.083	0.003 0.029 0.029	0.85 0.005 0.004
H3c	Cortical volume of the middle temporal gyrus and bank of the superior temporal sulcus	Indirect Direct Total	PT → ROI → DS PT → DS PT → DS	0.002 0.220 0.222	0.005 0.032 0.031	0.709 < 0.001 < 0.001	-0.004 0.087 0.083	0.004 0.029 0.029	0.362 0.003 0.004
H3d	Cortical volume of the middle frontal gyrus and postcentral gyrus	Indirect Direct Total	PT → ROI → DS PT → DS PT → DS	0.004 0.217 0.221	0.005 0.032 0.031	0.433 < 0.001 < 0.001	0.003 0.08 0.083	0.004 0.03 0.029	0.374 0.007 0.004
H3e	Fractional anisotropy of the cortico-striatal tract and corpus collosum	Indirect Direct Total	PT → ROI → DS PT → DS PT → DS	0.001 0.266 0.227	0.003 0.033 0.032	0.826 < 0.001 < 0.001	-0.003 0.076 0.073	0.003 0.031 0.03	0.278 0.013 0.016
H3f	Sulcal depth of the pars orbitalis	Indirect Direct Total	PT → ROI → DS PT → DS PT → DS	0.000 0.222 0.222	0.002 0.031 0.031	0.925 < 0.001 < 0.001	-0.002 0.085 0.084	0.002 0.029 0.002	0.371 0.004 0.371
H3g	Cortical volume of the ventral diencephalon	Indirect Direct Total	PT → ROI → DS PT → DS PT → DS	0.000 0.222 0.222	0.001 0.031 0.031	0.888 < 0.001 < 0.001	-0.001 0.084 0.083	0.001 0.029 0.029	0.668 0.004 0.004

### 4.4 Hypothesis 3 — regional brain measures

Our third hypothesis investigated whether regional brain structural measures at Year 2 (identified via our pilot analyses and listed in Table 1) mediated the association between earlier pubertal timing at Year 1 and later youth depressive symptoms at Year 3. As shown in Table 5, for both females and males, our results did not find support for our hypotheses due to an absence of an indirect effect (Females: ß range: −0.008 to 0.004 [IRR range = 0.99–1.00], *p* range: 0.05–0.89; Males: ß range: −0.004 to 0.003 [IRR range = 0.99–1.00], *p* range: 0.27–0.85).

All model statistics and Mplus outputs for these analyses, including single mediator model results (not reported in Table 5), can be found in the https://osf.io/rw3s6/?view_only=39ce180796ad4688bdbcf80563a726b5. Supplementary Data.

### 4.5 Exploratory analyses related to Hypothesis 2 & 3

We conducted exploratory analyses to identify whether any other brain structural measures (beyond those identified in the pilot analyses) mediated the association between earlier pubertal timing and depressive symptoms. Full details of these exploratory analyses can be found in the Supplementary Information. In brief, we first examined associations between pubertal timing (Year 1) and brain structure (Year 2), and between brain structure (Year 2) and youth depressive symptoms (Year 3). Lower volume of the accumbens area was the only brain measure found to be associated with *both* pubertal timing and depressive symptoms. Thus, we tested for mediation using the same methods employed in our confirmatory analyses.

#### 4.5.1 Brain structural associations with pubertal timing

**Fig. 6:**
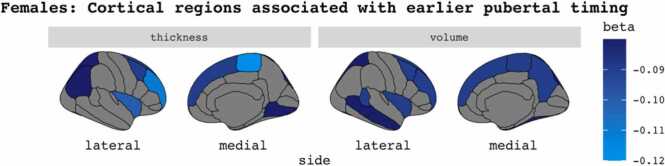
Exploratory analyses results: Significant cortical associations with earlier pubertal timing in female youth. p_FDR_ ≤ 0.001.

For females, earlier pubertal timing was associated with reduced global cortical thickness (ß = −0.10; *p*_FDR_ = 4.4 ×10^-5^) and global cortical volume (ß = −0.09; *p*_FDR_ = 1.3 ×10^-5^). Regionally, earlier pubertal timing was associated with reduced cortical thickness and volume in temporal, frontal and parietal regions (ß range: −0.12 to −0.08; *p*_FDR_ range: 2.2 ×10^-6^ to 0.0008). See Fig. 6.

For males, earlier pubertal timing was not significantly associated with global brain measures. The only regional brain measure that demonstrated a significant association with earlier pubertal timing was increased volume of the ventral diencephalon (ß = −0.07; *p*_FDR_ = 0.001).

#### 4.5.2 Brain structural associations with depressive symptoms

For females, no significant associations were found between global brain measures and depressive symptoms. However, we report several significant regional associations with depressive symptoms, namely, reduced volume in the accumbens area (ß = −0.105 [IRR = 0.90], *p*_FDR_ = 0.024, increased sulcal depth in the bank of the superior temporal sulcus (ß = 0.133 [IRR = 1.14], *p*_FDR_ = 0.003) and precuneus (ß = 0.12 [IRR = 1.12], *p*_FDR_ = 0.020), as well as increased MD in the inferior fronto-occipital fasciculus (ß = 0.11 [IRR = 1.12], *p*_FDR_ = 0.050).

For males, we did not find any significant associations between global brain measures and depression symptoms. Regionally, depressive symptoms were associated with reduced volume in the accumbens area (ß = −0.10 [IRR = 0.90], *p*_FDR_ = 0.012), pallidum (ß = −0.08 [IRR = 0.92], *p*_FDR_ = 0.052), and thalamus (ß = −0.08 [IRR = 0.92], *p*_FDR_ = 0.056), as well as reduced surface area in the medial orbitofrontal gyrus (ß = −0.11 [IRR = 0.89], *p*_FDR_ = 0.029).

#### 4.5.3 Testing accumbens area volume as a mediator

**Fig. 7:**
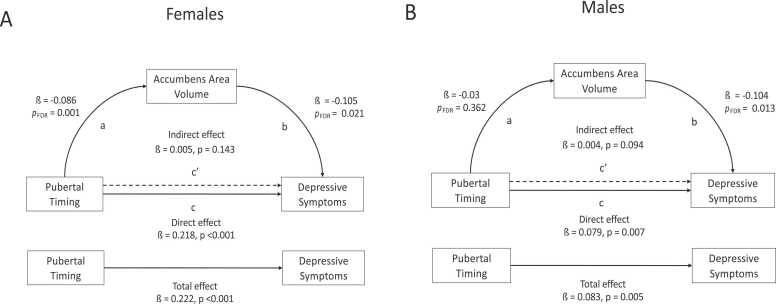
Exploratory analyses results: Mediation paths and statistics for main effect of pubertal timing and depressive symptoms, mediated through accumbens area volume. Results for females are shown in (A) and males are shown in (B).

For both females and males, we did not find any evidence of a mediating effect of accumbens area volume on the association between earlier pubertal timing and increased depressive symptoms (Females indirect effect: ß = 0.005, *p* = 0.14; Fig. 7a; Males indirect effect: ß = 0.004, *p* = 0.09; Fig. 7b).

### 4.6 Missing data

Compared to complete case analysis for H1, similar effect sizes were observed when missing data was imputed using MICE. Details of our imputation methods and results can be found in the Supplementary Information.

### 4.7 Sensitivity analyses

In our analysis of change in depressive symptoms over time and pubertal timing, we report a significant relationship in females only (Base model: ß = 0.17 [IRR = 1.17]; *p* < 0.001; fully adjusted model: ß = 0.16 [IRR = 1.73]; *p* < 0.001). Base and fully adjusted models for females and males are reported in the Supplementary Information. In our sensitivity analyses, we also found similar results to our main analyses when adding a population stratification weight to our models. See Supplementary Information for further details.

## 5 Discussion

In the present study, we investigated whether earlier pubertal timing was associated with an increased risk for later depressive symptoms in adolescence, and whether certain a priori brain structural measures mediated this association, in a large, demographically diverse sample of youth. We found that earlier pubertal timing when youth were aged 10–11 years was significantly associated with increased depressive symptoms two years later, when youth were aged 12–13 years. The observed association was stronger for female adolescents compared to males and importantly, this association did not remain significant in males when controlling for other factors associated with depression risk. In females, earlier pubertal timing was also related to worsening depressive symptoms over time. Thus, the first hypothesis of this registered report was partially supported. Regarding Hypothesis 2 & 3, the hypothesised brain structural measures were not found to mediate the association between earlier pubertal timing and later depressive symptoms. Although our exploratory analyses demonstrated significant brain structural associations with earlier pubertal timing and youth depressive symptoms, we also did not find evidence of brain structural mediation when examining regions beyond those specified a priori.

The current results advance our understanding of how pubertal timing relates to brain structural maturation and depression risk beyond age-related changes using one of the largest available samples to date. Our findings suggest that while a robust association exists between earlier pubertal timing and increased depressive symptoms, particularly for females, brain structure did not mediate this association in our analyses over these time points. These results highlight the need to consider additional biological factors (e.g., genetics), other brain metrics (e.g., brain function, brain age gap estimates (AGE)) and socio-environmental risk factors when examining the association between earlier pubertal timing and increased depression risk, and the differential impact they may have across sexes and time. Given the significant burden of depression in adolescence and beyond, further longitudinal research is urgently needed so that we can better understand how to support young people as they navigate this formative developmental transition.

### 5.1 Earlier pubertal timing is associated with later youth depressive symptoms

A substantive body of evidence has demonstrated that youth that begin puberty ahead of their peers are at an increased risk of psychopathology, including depression. Using one of the largest sample sizes to date (N = ∼ 5300), our findings extend prior work and emphasise the detrimental effects that earlier pubertal maturation can have on depression risk in adolescence. Our results are broadly consistent with the maturation disparity hypothesis which posits that both early maturing males and females are at an increased risk for mental health difficulties during adolescence, due to an incongruency in their physical, cognitive, social, and emotional development (Brooks-Gunn et al., 1985, Ge et al., 2001, Ge and Natsuaki, 2009). Our findings are similar to earlier meta-analytic work (Ullsperger and Nikolas, 2017) and recent findings from the ABCD Study (McNeilly et al., 2022) in that we also found a significant small effect size for the association between earlier pubertal timing and internalising difficulties. However, we additionally report a greater magnitude of effect for early maturing females compared to males, suggesting that female youth that hit puberty ahead of their peers are particularly at risk for mood difficulties.

Ullsperger and Nikolas (2017) did not find evidence of a female-specific vulnerability for internalising difficulties in early maturing youth. A direct comparison of results is difficult here given that we used a depression-specific outcome measure, whereas Ullsperger and Nikolas (2017) used a broader index of internalising difficulties that comprised distress, fear, and eating pathology. Of note, when the authors examined male and female samples separately, they found a significant association between pubertal timing and distress for females but not males, although in the whole group analysis, a sex moderation effect was not found. Using baseline ABCD data, McNeilly et al. (2022) report similar effect sizes for male and female youth aged 9–10 years. However, as the authors note, the female-specific vulnerability for internalising difficulties does not emerge until the age of 12 years or older, which may explain why we observed a greater magnitude of effect for females in our study which used later follow up ABCD data (youth aged 12–13 years when depression was measured).

Importantly, at the time pubertal development was assessed in our study (youth aged 10–11 years), most males were pre-pubertal or in the early stages of puberty, while the females exhibited a much broader spread of pubertal maturation. Thus, the greater magnitude of effect we observe for females could reflect a temporal effect. That is, the distress associated with the experience of maturing ahead of your peers may not manifest straight away and could become apparent in male youth at a later timepoint when they are more pubertally mature. Further, our sensitivity analyses revealed that when controlling for earlier depressive symptoms, earlier pubertal timing was associated with the worsening of symptoms (between ages 10–11 years and 12–13 years) in females but not males. This may reflect the significantly higher incidence of depressive symptoms in females compared to males in the current sample.

It may also be that the inconsistent findings in the current literature reflect a general direct effect between earlier pubertal timing and depressive symptoms that is comprised of sex-specific vulnerabilities. For example, previous research has shown that ethnicity, life stress, and cognitive processes (e.g., rumination) moderate the risk of earlier pubertal timing for later psychopathology according to sex (Alloy et al., 2016, Hamilton et al., 2014). Indeed, the findings of the current study underscore the importance of considering this nuance: the main effect between earlier pubertal timing and increased depressive symptoms was no longer significant for males only when additional socio-demographic factors (e.g., BMI) were controlled for. This provides further evidence that while early maturing youth are at an increased risk for depression in adolescence, there may be sex-specific biological and social/environmental mechanisms that influence this risk. Future research that embraces a biopsychosocial conceptual framework (e.g., Affective, Biological and Cognitive (ABC) model of depression; Hyde et al., 2008) is needed to refine existing theories so that they better reflect the complex interplay of risk (and resilience promoting) factors that underpin the association between pubertal timing and psychopathology in adolescence and how this may vary across sexes (Ullsperger and Nikolas, 2017).

Our exploratory analyses found that both adrenarcheal and gonadarcheal timing were associated with depressive symptoms in females, consistent with prior findings (Barendse et al., 2021). However, for males, we report that the observed association between early pubertal timing and later depressive symptoms was driven by adrenarcheal aspects of pubertal maturation. As mentioned, while the males and females in the ABCD sample are the same chronological age, there are marked differences in their pubertal progression. Thus, multiple timepoints of data, where there is similar variation in pubertal maturation for both sexes, are needed to properly investigate sex differences in pubertal timing, as well as tempo, and their relation to depression risk. These data will soon be available from the ABCD Study and the results from the current study have laid a strong foundation for this future work.

### 5.2 Brain structure does not mediate the association between earlier pubertal timing and later depressive symptoms

We did not find that cortical, subcortical, or white matter microstructural measures mediated the association between earlier pubertal timing and increased depressive symptoms in adolescents, in both our confirmatory and exploratory mediation analyses. These results suggest that although pubertal timing was associated with alterations in brain morphology above and beyond age-related changes, and there were associations between brain features and depression, these brain structural features did not mediate increased risk for later depressive symptoms in this sample (9–13 years). The present findings highlight that much work remains in our effort to better understand what it is about the experience of developing ahead of your peers that confers vulnerability to depression in adolescence.

This vulnerability is likely comprised of multiple and interacting biological and socio-environmental factors that exert varying degrees of risk across time and individuals. How other neuroimaging features, such as brain function and multi-modal brain metrics (e.g., brainAGE), relate to depression risk in early maturing youth remain underexplored (Pfeifer and Allen, 2021). It may be that certain brain features (e.g., structural and/or functional) mediate the association between earlier pubertal timing and depression in some but *not* all youth, and such associations may only exist at certain developmental windows (e.g., in mid adolescence). Shifting our focus from group-level analyses to a person-centred longitudinal approach, which will soon be possible in ABCD with the release of multiple timepoints (>3) of imaging data, will allow us to better assess null findings, such as those in the current study.

Access to large-scale, longitudinal neuroimaging data in developmental samples will undoubtedly create many exciting avenues for future research. However, there has been much debate about how meaningful the small effect sizes consistently reported in large-scale neuroimaging studies are and crucially, what they really add to our ability to predict developmental outcomes when compared to clinical and psychosocial data (Dick et al., 2021). Given the complex web of interactions that shape development, it is perhaps unsurprising that an individual measure (e.g., global cortical volume or the volume of the nucleus accumbens) only explains a small amount of variance in our outcome of interest (e.g., depression). If we are ever to use an “ecological neuroscience” approach that combines neuroimaging with other biological and psycho-social factors to reliably predict developmental outcomes, we need to be able to do so at the level of the individual (Ferschmann et al., 2022). The availability of longitudinal multimodal data paired with the advent of advanced analytic methods holds great promise in answering such research questions and should be explored in subsequent work.

Our exploratory work extends existing research by examining brain structural associations with pubertal timing in one of the largest samples to date (N = ∼5000). Crucially, our analyses pertain to brain structural associations with pubertal timing specifically which is distinct from examining pubertal development controlling for age, a distinction that is often overlooked in the extant literature. We found that earlier pubertal timing was associated with lower cortical volume and thickness both globally and regionally in frontal, temporal, and parietal regions. Our results also demonstrated that a decrease in the volume of the nucleus accumbens was related to earlier pubertal timing. The current findings are consistent with prior work that has used both physical and hormonal puberty measures (Goddings et al., 2014, Goddings et al., 2019, Vijayakumar et al., 2018). Although a positive association between pubertal timing and FA has been reported previously (Herting et al., 2012, Peper et al., 2015), we did not find that white matter microstructure was associated with pubertal timing in our sample. Importantly, the above findings pertain to female youth only and the only brain structural association with pubertal timing observed in males was increased volume of the ventral diencephalon. Due to differences in the age of puberty onset between sexes, and the age range (9–13 years) of the current sample, we may not yet see puberty-related brain structural effects in males. Longitudinal work is needed to further understand how pubertal timing affects brain structural development over time, which will help elucidate whether the sex differences observed in the current study attenuate as the pubertal stages of females and males align.

Our earlier work (Shen et al., 2021), and that of others (Schmaal et al., 2017), has demonstrated that global and regional alterations in cortical and white matter microstructural measures are associated with depression in adolescence. The results of the current study are broadly aligned with these earlier findings such that we see differences in some temporal, parietal and frontal regions, as well as in fronto-occipital white matter tracts, with a consistent directionality of effects. We also report alterations in subcortical areas such as reduced volume of the nucleus accumbens (in both sexes), pallidum, and thalamus (males only). Together with previous work, the current findings can inform future longitudinal research that models individual differences in brain development. This work is needed to determine how cross-sectional depression-related imaging features relate to patterns of brain maturation over time (i.e., do they reflect accelerated or delayed development) and how they vary across individuals (Becht and Mills, 2020).

### 5.3 Limitations and future directions

Although our study benefits from a large sample size and a mediation analysis that used temporally distinct timepoints, there are important limitations to consider. Firstly, our measure of pubertal timing is cross-sectional and based on parent-report. Using a cross-sectional measure of pubertal timing precludes investigating whether the rate of change in pubertal maturation (i.e., tempo) matters for developmental outcomes. For example, if a young person is an “early developer” at the start of puberty but “on-time” by mid-puberty, how does this relate to depression risk. Longitudinal data with repeated measures of pubertal development will be essential in answering such research questions. Additionally, we prioritised parent-report of youth pubertal development (over youth self-report) because youth have been found to over-report their pubertal development in the early stages of adolescence (Schlossberger et al., 1992). There was also a large number of “I don’t know” responses in the early waves of ABCD puberty data collection (Cheng et al., 2021). Nonetheless, adolescent report may better capture the more intimate body changes associated with puberty, especially in the later pubertal stages (Dorn et al., 1990). Importantly, self- (and parent-) report measures of pubertal development assess the outcome of prolonged systemic hormonal effects and thus are limited in their ability to make inferences about the biological mechanisms relating pubertal timing to neurodevelopment (Goddings et al., 2019). Due to the availability of multi-modal puberty and imaging data, ABCD is well-positioned to advance this line of research and could build upon emerging multi-verse analysis methods (Barendse et al., 2021).

There are a number of measurement error considerations related to the PDS that warrant attention. For example, PDS responses mix rate of change and stage, such that someone experiencing rapid pubertal changes (i.e., tempo) might be more likely to select the response “definitely underway” compared to someone with a more protracted pubertal development. Similarly, the yearly interval between assessments in ABCD may mean that aspects of pubertal development may be described as “complete” even though further changes could occur later. As longitudinal data become available in ABCD, such considerations should be explored so that we can map and interpret patterns of pubertal maturation as accurately as possible, while acknowledging the limitations of the measures available. Akin to the limitations associated with parent-report of pubertal development, examining adolescents’ self-report of depressive symptoms, and how this relates to parent report, is an important consideration for future work (Shen et al., 2021). Additionally, our use of parent report for both the predictor and outcome measures could have resulted in shared methods variance. Adopting a multi-informant approach will help address this limitation in subsequent research.

Although the temporal distance between pubertal timing, brain structure, and youth depression measures was a strength of the analyses undertaken in the current study, due to the varying availability of follow-up data, we did not examine any changes in our variables of interest between timepoints. Moreover, a brain structure previously found to mediate the association between earlier pubertal timing and depression in adolescence, namely, the pituitary gland (Whittle et al., 2012), was not available in the brain parcellations provided in the ABCD curated data release and was thus not tested as a potential mediator. We recommend using the raw ABCD imaging data to test the pituitary gland specifically as a mediator in further research. Further, subsequent work should also reflect the temporality inherent to development by examining domains such as pubertal tempo (the rate at which pubertal development occurs), and how this relates to both brain structural and functional changes across adolescence, as well as depressive symptom trajectories.

Charting individual differences in development has gained increasing attention in recent times but longitudinal studies with multiple timepoints are necessary to generate developmental pathways (Bethlehem et al., 2022, Mills et al., 2021). For example, do differences in pubertal timing represent a stable risk factor that predicts the emergence of depression or do other factors (e.g., early life stress, loneliness) exert varying degrees of influence during adolescence (Colich and McLaughlin, 2022). Given that knowledge gaps still exist in our understanding of normative brain development during adolescence (particularly in terms of brain function), global neuroimaging metrics, such as “BrainAGE” may better capture deviations (e.g., acceleration) from typical neuromaturation, and on what scale this occurs (globally, or in particular brain networks) (Colich and McLaughlin, 2022, Popescu et al., 2021). Emerging research has already begun to explore such questions by examining brain maturation and puberty in early adolescence using deep learning brain age prediction models (Holm et al., 2023).

### 5.4 Conclusion

The current study makes a significant contribution to our understanding of how pubertal maturation relates to youth depression by directly testing whether brain structural features mediate this association, which had not been previously examined in a sample of this size and with multiple timepoints of data (Pfeifer and Allen, 2021). We found that while early maturing youth, particularly females, were at an increased risk for depression, brain structure was not found to mediate the observed association in either sex. Central to the new conceptual model proposed by Pfeifer and Allen (2021) is the consideration of neural, social, and pubertal processes simultaneously and how they co-evolve and interact over time. Therefore, the work undertaken in this registered report can be used a framework for future studies, whose design should reflect the complex interplay of these processes as much as possible. Given the complexities of development, “team-science” and “open-science” practices will allow us to better understand the aspects of pubertal and brain development that contribute to adolescent mental health vulnerabilities in a reproducible and collaborative manner (Zanolie et al., 2022). Such efforts will be crucial to reaching our collective goal of creating an environment that gives young people every chance to flourish in their development.

## 6 Data access

N.M and all co-authors self-certify that they did not observe any of the statistical models outlined in the confirmatory analysis until after the in-principle acceptance was issued. See Supplementary Information for full information on data access. All scripts (R and Mplus) used in this registered report are available on the GitHub repository for this project: https://github.com/niamhmacsweeney/ABCD_puberty_depression.

## Data Availability

The code used for the analyses in this registered report can be found at https://github.com/niamhmacsweeney/ABCD_puberty_depression.git.
