# Supplementary material for: The role of brain structure in the association between pubertal timing and depression risk in an early adolescent sample (the ABCD Study®): A registered report
Source: Dev Cogn Neurosci. 2023 Feb 24;60:101223. doi: 10.1016/j.dcn.2023.101223 (PMC10009199; doi:10.1016/j.dcn.2023.101223)
Supplement: Supplementary file 1 — Supplementary material [file mmc1.docx]

Supplementary Information

The role of brain structure in the association between pubertal timing and depression risk in an early adolescent sample (the ABCD Study®): A registered report

MacSweeney et al.

Table of Contents

[Main analyses 3](#_Toc126332322)

[Hypothesis 1 — Earlier pubertal timing is associated with later depression symptoms 3](#_Toc126332323)

[Exploratory analyses 6](#_Toc126332324)

[Hypothesis 1: Gonadal and adrenal pubertal timing measures 6](#_Toc126332325)

[Hypotheses 2 & 3: Whole brain exploratory analyses 15](#_Toc126332326)

[Sensitivity Analyses 17](#_Toc126332327)

[Hypothesis 1: Multiple imputation of missing outcome and covariate data 17](#_Toc126332328)

[Hypothesis 1: Controlling for earlier youth depression 20](#_Toc126332329)

[Hypothesis 1: Population weight raked propensity score 24](#_Toc126332330)

[Pilot analyses 28](#_Toc126332331)

[Statistical model specifications 28](#_Toc126332332)

[Pilot results 30](#_Toc126332333)

[Data access 34](#_Toc126332334)

[References 35](#_Toc126332335)

# Main analyses

## Hypothesis 1 — Earlier pubertal timing is associated with later depression symptoms

The complete model output, including incidence rate ratios, standard errors, and p-values for the predictor and all covariates across the base and fully adjusted models are reported in Table S1 (females) and Table S2 (males).

| **Females: Effect of pubertal timing on youth depression** | | | | | | |
| --- | --- | --- | --- | --- | --- | --- |
|  | **Base** | | | **Full** | | |
| *Predictors* | *IRR* | *SE* | *P-Value* | *IRR* | *SE* | *P-Value* |
| (Intercept) | 0.821 | 0.032 | **<0.001** | 0.636 | 0.172 | 0.093 |
| Pubertal timing | 1.313 | 0.041 | **<0.001** | 1.220 | 0.040 | **<0.001** |
| Age | 1.111 | 0.034 | **0.001** | 1.097 | 0.034 | **0.002** |
| Race: Black | 0.630 | 0.073 | **<0.001** | 0.723 | 0.088 | **0.007** |
| Race: Asian | 0.854 | 0.187 | 0.471 | 1.032 | 0.222 | 0.884 |
| Race: AIAN/NHPI | 0.961 | 0.360 | 0.915 | 0.884 | 0.328 | 0.739 |
| Race: Other | 1.299 | 0.213 | 0.110 | 1.497 | 0.246 | **0.014** |
| Race: Mixed | 1.217 | 0.116 | **0.040** | 1.138 | 0.107 | 0.169 |
| BMI |  |  |  | 1.105 | 0.036 | **0.002** |
| Household income: $5,000-$11,999 |  |  |  | 1.094 | 0.371 | 0.792 |
| Household income: $12,000-$15,999 |  |  |  | 0.850 | 0.297 | 0.642 |
| Household income: $16,000-$24,999 |  |  |  | 1.379 | 0.430 | 0.303 |
| Household income: $25,000-$34,999 |  |  |  | 1.107 | 0.331 | 0.735 |
| Household income: $35,000-$49,999 |  |  |  | 1.339 | 0.383 | 0.307 |
| Household income: $50,000-$74,999 |  |  |  | 1.300 | 0.364 | 0.349 |
| Household income: $75,000-$99,999 |  |  |  | 1.245 | 0.349 | 0.433 |
| Household income: $100,000-$199,999 |  |  |  | 1.338 | 0.367 | 0.288 |
| Household income: >$200,000 |  |  |  | 1.255 | 0.358 | 0.427 |
| Parent depressive symptoms |  |  |  | 1.500 | 0.046 | **<0.001** |
| **Random Effects** | | | | | | |
| σ^2^ | 0.81 | | | 0.81 | | |
| τ_00_ | 1.29 _rel_family_id_ | | | 1.12 _rel_family_id_ | | |
|  | 0.00 _site_id_y1_ | | | 0.00 _site_id_y1_ | | |
| N | 21 _site_id_y1_ | | | 21 _site_id_y1_ | | |
|  | 2155 _rel_family_id_ | | | 2105 _rel_family_id_ | | |
| Observations | 2491 | | | 2426 | | |
| Marginal R^2^ / Conditional R^2^ | 0.111 / NA | | | 0.252 / NA | | |

Table S1 — Females: Base and Fully adjusted models with associated statistics for effect of earlier pubertal timing on later depressive symptoms. Note: IRR = incidence rate ratio. AIAN/NHPI = AIAN/NHPI = American Indian/Alaska Native/Native Hawaiian and other Pacific Islander.

| **Males: Effect of pubertal timing on youth depression** | | | | | | |
| --- | --- | --- | --- | --- | --- | --- |
|  | **Base** | | | **Full** | | |
| *Predictors* | *IRR* | *SE* | *P-Value* | *IRR* | *SE* | *P-Value* |
| (Intercept) | 0.738 | 0.027 | **<0.001** | 0.864 | 0.178 | 0.478 |
| Pubertal timing | 1.088 | 0.033 | **0.006** | 1.045 | 0.032 | 0.151 |
| Age | 1.041 | 0.031 | 0.167 | 1.025 | 0.029 | 0.397 |
| Race: Black | 0.685 | 0.083 | **0.002** | 0.671 | 0.083 | **0.001** |
| Race: Asian | 0.586 | 0.134 | **0.019** | 0.735 | 0.167 | 0.175 |
| Race: AIAN/NHPI | 0.950 | 0.419 | 0.908 | 1.046 | 0.443 | 0.915 |
| Race: Other | 1.096 | 0.186 | 0.590 | 1.036 | 0.173 | 0.834 |
| Race: Mixed | 1.165 | 0.118 | 0.130 | 1.087 | 0.106 | 0.391 |
| BMI |  |  |  | 1.126 | 0.033 | **<0.001** |
| Household income: $5,000-$11,999 |  |  |  | 0.677 | 0.198 | 0.182 |
| Household income: $12,000-$15,999 |  |  |  | 0.722 | 0.232 | 0.311 |
| Household income: $16,000-$24,999 |  |  |  | 1.051 | 0.257 | 0.838 |
| Household income: $25,000-$34,999 |  |  |  | 0.909 | 0.218 | 0.692 |
| Household income: $35,000-$49,999 |  |  |  | 0.989 | 0.222 | 0.962 |
| Household income: $50,000-$74,999 |  |  |  | 0.857 | 0.186 | 0.476 |
| Household income: $75,000-$99,999 |  |  |  | 0.839 | 0.182 | 0.419 |
| Household income: $100,000-$199,999 |  |  |  | 0.819 | 0.174 | 0.347 |
| Household income: >$200,000 |  |  |  | 0.840 | 0.189 | 0.436 |
| Parent depressive symptoms |  |  |  | 1.562 | 0.045 | **<0.001** |
| **Random Effects** | | | | | | |
| σ^2^ | 0.87 | | | 0.87 | | |
| τ_00_ | 1.28 _rel_family_id_ | | | 1.04 _rel_family_id_ | | |
|  | 0.00 _site_id_y1_ | | | 0.00 _site_id_y1_ | | |
| N | 21 _site_id_y1_ | | | 21 _site_id_y1_ | | |
|  | 2412 _rel_family_id_ | | | 2369 _rel_family_id_ | | |
| Observations | 2752 | | | 2703 | | |
| Marginal R^2^ / Conditional R^2^ | 0.030 / NA | | | 0.224 / NA | | |

Table S2 — Males: Base and Fully adjusted models with associated statistics for effect of earlier pubertal timing on later depressive symptoms. Note: IRR = incidence rate ratio. AIAN/NHPI = AIAN/NHPI = American Indian/Alaska Native/Native Hawaiian and other Pacific Islander.

# Exploratory analyses

## Hypothesis 1: Gonadal and adrenal pubertal timing measures

To derive gonadal and adrenal pubertal timing measures, we first calculated a gonadal Pubertal Developmental Scale (PDS) score and adrenal PDS score. As previously described by Shirtcliff et al., 2009, and adopted by others using the ABCD puberty data (e.g., Herting et al., 2021), a gonadal PDS score was generated for females by averaging growth spurt, breast development and menarche PDS items (variables: pds_1_p, pds_f4_p, pds_f5b_p), and for males by averaging growth spurt, deepening of voice, and facial hair PDS items (variables: pds_1_p, pds_m4_p, pds_m5_p). Adrenal PDS scores were calculated for both sexes by averaging pubic, body hair and skin changes PDS items (pds_2_p, pds_3_p). A pubertal timing score for adrenal and gonadal measures was then obtained by regressing the adrenal and gonadal PDS scores on age, and using the residual obtained as the timing measure. This was done for males and females separately.

Independent models for gonadal and adrenal pubertal timing are presented in Tables S3 & S4 for females and S5 & S6 for males.

As further post-hoc analysis, we included both GT and AT scores in the same model (base model specification) to investigate whether one aspect of pubertal development was significantly associated with later youth depression, above and beyond the other. First, Spearman’s rank correlation was computed to assess the relationship between AT and GT measures (Females: Spearman's 𝜌 = 0.61; Males: Spearman's 𝜌 = 0.44). Model specifications:$youth depression \sim gonadal timing+adrenal timing+age+race+ 1\left| site ID+1 \right| family ID$.

Our results suggest that, in females, both GT and AT contribute significantly to the association between earlier pubertal timing and youth depression (GT, controlling for AT, ß = 0.18 [IRR = 1.19]; *p* < 0.001; AT: ß = 0.12 [IRR = 1.13]; p = 0.001). We note here that the effect size for GT is slightly larger than AT. For males, our results suggest that the association between earlier pubertal timing and later youth depression is being driven by AT rather than GT (GT controlling for AT, ß = -0.006 [IRR = 0.994]; *p* = 0.85; AT: ß = 0.10 [IRR = 1.11]; *p* = 0.003). See Table S7 (females) and S8 (males).

| **Females: Effect of adrenal pubertal timing on youth depression** | | | | | | |
| --- | --- | --- | --- | --- | --- | --- |
|  | **Base** | | | **Full** | | |
| *Predictors* | *IRR* | *SE* | *P-Value* | *IRR* | *SE* | *P-Value* |
| (Intercept) | 0.814 | 0.032 | **<0.001** | 0.654 | 0.176 | 0.114 |
| Adrenal pubertal timing | 1.259 | 0.040 | **<0.001** | 1.179 | 0.038 | **<0.001** |
| Age | 1.114 | 0.034 | **<0.001** | 1.095 | 0.033 | **0.003** |
| Race: Black | 0.667 | 0.077 | **<0.001** | 0.734 | 0.089 | **0.011** |
| Race: Asian | 0.881 | 0.193 | 0.563 | 1.066 | 0.229 | 0.766 |
| Race: AIAN/NHPI | 1.023 | 0.382 | 0.951 | 0.900 | 0.333 | 0.775 |
| Race: Other | 1.388 | 0.227 | **0.045** | 1.545 | 0.253 | **0.008** |
| Race: Mixed | 1.248 | 0.119 | **0.020** | 1.151 | 0.108 | 0.133 |
| BMI |  |  |  | 1.131 | 0.036 | **<0.001** |
| Household income: $5,000-$11,999 |  |  |  | 1.074 | 0.364 | 0.834 |
| Household income: $12,000-$15,999 |  |  |  | 0.843 | 0.294 | 0.626 |
| Household income: $16,000-$24,999 |  |  |  | 1.372 | 0.427 | 0.310 |
| Household income: $25,000-$34,999 |  |  |  | 1.089 | 0.325 | 0.775 |
| Household income: $35,000-$49,999 |  |  |  | 1.308 | 0.373 | 0.347 |
| Household income: $50,000-$74,999 |  |  |  | 1.273 | 0.355 | 0.387 |
| Household income: $75,000-$99,999 |  |  |  | 1.210 | 0.337 | 0.495 |
| Household income: $100,000-$199,999 |  |  |  | 1.290 | 0.352 | 0.351 |
| Household income: >$200,000 |  |  |  | 1.197 | 0.340 | 0.528 |
| Parent depressive symptoms |  |  |  | 1.501 | 0.046 | **<0.001** |
| **Random Effects** | | | | | | |
| σ^2^ | 0.81 | | | 0.81 | | |
| τ_00_ | 1.29 _rel_family_id_ | | | 1.11 _rel_family_id_ | | |
|  | 0.00 _site_id_y1_ | | | 0.00 _site_id_y1_ | | |
| N | 21 _site_id_y1_ | | | 21 _site_id_y1_ | | |
|  | 2155 _rel_family_id_ | | | 2105 _rel_family_id_ | | |
| Observations | 2491 | | | 2426 | | |
| Marginal R^2^ / Conditional R^2^ | 0.092 / NA | | | 0.246 / NA | | |

Table S3 — Females: Adrenal pubertal timing and youth depression: Base and Fully adjusted models with associated statistics. Note: IRR = incidence rate ratio. AIAN/NHPI = AIAN/NHPI = American Indian/Alaska Native/Native Hawaiian and other Pacific Islander.

| **Females: Effect of gonadal pubertal timing on youth depression** | | | | | | |
| --- | --- | --- | --- | --- | --- | --- |
|  | **Base** | | | **Full** | | |
| *Predictors* | *IRR* | *SE* | *P-Value* | *IRR* | *SE* | *P-Value* |
| (Intercept) | 0.817 | 0.032 | **<0.001** | 0.628 | 0.170 | 0.085 |
| Gonadal pubertal timing | 1.281 | 0.039 | **<0.001** | 1.191 | 0.038 | **<0.001** |
| Age | 1.110 | 0.034 | **0.001** | 1.096 | 0.034 | **0.003** |
| Race: Black | 0.646 | 0.075 | **<0.001** | 0.737 | 0.089 | **0.012** |
| Race: Asian | 0.834 | 0.183 | 0.410 | 1.004 | 0.218 | 0.985 |
| Race: AIAN/NHPI | 0.951 | 0.358 | 0.894 | 0.871 | 0.324 | 0.710 |
| Race: Other | 1.267 | 0.209 | 0.150 | 1.467 | 0.242 | **0.020** |
| Race: Mixed | 1.214 | 0.117 | **0.044** | 1.133 | 0.107 | 0.184 |
| BMI |  |  |  | 1.114 | 0.036 | **0.001** |
| Household income: $5,000-$11,999 |  |  |  | 1.105 | 0.376 | 0.769 |
| Household income: $12,000-$15,999 |  |  |  | 0.870 | 0.305 | 0.691 |
| Household income: $16,000-$24,999 |  |  |  | 1.391 | 0.435 | 0.291 |
| Household income: $25,000-$34,999 |  |  |  | 1.131 | 0.340 | 0.682 |
| Household income: $35,000-$49,999 |  |  |  | 1.351 | 0.388 | 0.295 |
| Household income: $50,000-$74,999 |  |  |  | 1.315 | 0.369 | 0.330 |
| Household income: $75,000-$99,999 |  |  |  | 1.258 | 0.353 | 0.413 |
| Household income: $100,000-$199,999 |  |  |  | 1.348 | 0.371 | 0.277 |
| Household income: >$200,000 |  |  |  | 1.266 | 0.362 | 0.409 |
| Parent depressive symptoms |  |  |  | 1.506 | 0.047 | **<0.001** |
| **Random Effects** | | | | | | |
| σ^2^ | 0.81 | | | 0.81 | | |
| τ_00_ | 1.30 _rel_family_id_ | | | 1.13 _rel_family_id_ | | |
|  | 0.00 _site_id_y1_ | | | 0.00 _site_id_y1_ | | |
| N | 21 _site_id_y1_ | | | 21 _site_id_y1_ | | |
|  | 2155 _rel_family_id_ | | | 2105 _rel_family_id_ | | |
| Observations | 2491 | | | 2426 | | |
| Marginal R^2^ / Conditional R^2^ | 0.099 / NA | | | 0.247 / NA | | |

Table S4 — Females: Gonadal pubertal timing and youth depression: Base and Fully adjusted models with associated statistics. Note: IRR = incidence rate ratio. AIAN/NHPI = AIAN/NHPI = American Indian/Alaska Native/Native Hawaiian and other Pacific Islander.

| **Males: Effect of adrenal pubertal timing on youth depression** | | | | | | |
| --- | --- | --- | --- | --- | --- | --- |
|  | **Base** | | | **Full** | | |
| *Predictors* | *IRR* | *SE* | *P-Value* | *IRR* | *SE* | *P-Value* |
| (Intercept) | 0.737 | 0.027 | **<0.001** | 0.848 | 0.176 | 0.425 |
| Adrenal pubertal timing | 1.105 | 0.033 | **0.001** | 1.055 | 0.032 | 0.078 |
| Age | 1.042 | 0.031 | 0.162 | 1.025 | 0.029 | 0.388 |
| Race: Black | 0.682 | 0.082 | **0.002** | 0.670 | 0.083 | **0.001** |
| Race: Asian | 0.591 | 0.135 | **0.021** | 0.737 | 0.168 | 0.180 |
| Race: AIAN/NHPI | 0.966 | 0.427 | 0.939 | 1.051 | 0.446 | 0.907 |
| Race: Other | 1.110 | 0.188 | 0.538 | 1.043 | 0.174 | 0.802 |
| Race: Mixed | 1.164 | 0.118 | 0.134 | 1.087 | 0.106 | 0.392 |
| BMI |  |  |  | 1.125 | 0.033 | **<0.001** |
| Household income: $5,000-$11,999 |  |  |  | 0.695 | 0.203 | 0.214 |
| Household income: $12,000-$15,999 |  |  |  | 0.733 | 0.236 | 0.333 |
| Household income: $16,000-$24,999 |  |  |  | 1.082 | 0.265 | 0.747 |
| Household income: $25,000-$34,999 |  |  |  | 0.924 | 0.222 | 0.741 |
| Household income: $35,000-$49,999 |  |  |  | 1.010 | 0.227 | 0.966 |
| Household income: $50,000-$74,999 |  |  |  | 0.872 | 0.190 | 0.530 |
| Household income: $75,000-$99,999 |  |  |  | 0.855 | 0.186 | 0.473 |
| Household income: $100,000-$199,999 |  |  |  | 0.833 | 0.177 | 0.391 |
| Household income: >$200,000 |  |  |  | 0.854 | 0.192 | 0.482 |
| Parent depressive symptoms |  |  |  | 1.560 | 0.045 | **<0.001** |
| **Random Effects** | | | | | | |
| σ^2^ | 0.87 | | | 0.87 | | |
| τ_00_ | 1.28 _rel_family_id_ | | | 1.04 _rel_family_id_ | | |
|  | 0.00 _site_id_y1_ | | | 0.00 _site_id_y1_ | | |
| ICC |  | | | 0.54 | | |
| N | 21 _site_id_y1_ | | | 21 _site_id_y1_ | | |
|  | 2412 _rel_family_id_ | | | 2369 _rel_family_id_ | | |
| Observations | 2752 | | | 2703 | | |
| Marginal R^2^ / Conditional R^2^ | 0.033 / NA | | | 0.116 / 0.597 | | |

Table S5 — Males: Adrenal pubertal timing and youth depression: Base and Fully adjusted models with associated statistics. Note: IRR = incidence rate ratio. AIAN/NHPI = AIAN/NHPI = American Indian/Alaska Native/Native Hawaiian and other Pacific Islander.

| **Males: Effect of gonadal pubertal timing on youth depression** | | | | | | |
| --- | --- | --- | --- | --- | --- | --- |
|  | **Base** | | | **Full** | | |
| *Predictors* | *IRR* | *SE* | *P-Value* | *IRR* | *SE* | *P-Value* |
| (Intercept) | 0.734 | 0.027 | **<0.001** | 0.862 | 0.178 | 0.472 |
| Gonadal pubertal timing | 1.044 | 0.032 | 0.154 | 1.021 | 0.031 | 0.487 |
| Age | 1.042 | 0.031 | 0.165 | 1.024 | 0.029 | 0.407 |
| Race: Black | 0.714 | 0.086 | **0.005** | 0.684 | 0.084 | **0.002** |
| Race: Asian | 0.581 | 0.133 | **0.018** | 0.732 | 0.167 | 0.171 |
| Race: AIAN/NHPI | 0.950 | 0.419 | 0.908 | 1.030 | 0.436 | 0.945 |
| Race: Other | 1.107 | 0.188 | 0.549 | 1.041 | 0.174 | 0.810 |
| Race: Mixed | 1.177 | 0.119 | 0.106 | 1.094 | 0.106 | 0.357 |
| BMI |  |  |  | 1.133 | 0.033 | **<0.001** |
| Household income: $5,000-$11,999 |  |  |  | 0.680 | 0.199 | 0.187 |
| Household income: $12,000-$15,999 |  |  |  | 0.723 | 0.232 | 0.312 |
| Household income: $16,000-$24,999 |  |  |  | 1.051 | 0.256 | 0.839 |
| Household income: $25,000-$34,999 |  |  |  | 0.913 | 0.219 | 0.703 |
| Household income: $35,000-$49,999 |  |  |  | 0.990 | 0.222 | 0.964 |
| Household income: $50,000-$74,999 |  |  |  | 0.858 | 0.186 | 0.479 |
| Household income: $75,000-$99,999 |  |  |  | 0.838 | 0.182 | 0.417 |
| Household income: $100,000-$199,999 |  |  |  | 0.818 | 0.173 | 0.342 |
| Household income: >$200,000 |  |  |  | 0.838 | 0.188 | 0.431 |
| Parent depressive symptoms |  |  |  | 1.563 | 0.045 | **<0.001** |
| **Random Effects** | | | | | | |
| σ^2^ | 0.87 | | | 0.87 | | |
| τ_00_ | 1.28 _rel_family_id_ | | | 1.04 _rel_family_id_ | | |
|  | 0.00 _site_id_y1_ | | | 0.00 _site_id_y1_ | | |
| N | 21 _site_id_y1_ | | | 21 _site_id_y1_ | | |
|  | 2412 _rel_family_id_ | | | 2369 _rel_family_id_ | | |
| Observations | 2752 | | | 2703 | | |
| Marginal R^2^ / Conditional R^2^ | 0.025 / NA | | | 0.223 / NA | | |

Table S6 — Males: Gonadal pubertal timing and youth depression: Base and Fully adjusted models with associated statistics. Note: IRR = incidence rate ratio. AIAN/NHPI = AIAN/NHPI = American Indian/Alaska Native/Native Hawaiian and other Pacific Islander.

| **Females: Effect of gonadal pubertal timing on youth depression, controlling for adrenal pubertal timing** | | | |
| --- | --- | --- | --- |
|  | **Base** | | |
| *Predictors* | *IRR* | *SE* | *P-Value* |
| (Intercept) | 0.821 | 0.032 | **<0.001** |
| Gonadal pubertal timing | 1.194 | 0.044 | **<0.001** |
| Adrenal pubertal timing | 1.132 | 0.044 | **0.001** |
| Age | 1.111 | 0.034 | **0.001** |
| Race: Black | 0.629 | 0.073 | **<0.001** |
| Race: Asian | 0.851 | 0.186 | 0.463 |
| Race: AIAN/NHPI | 0.973 | 0.364 | 0.941 |
| Race: Other | 1.294 | 0.212 | 0.117 |
| Race: Mixed | 1.216 | 0.116 | **0.041** |
| **Random Effects** | | | |
| σ^2^ | 0.81 | | |
| τ_00_ _rel_family_id_ | 1.29 | | |
| τ_00_ _site_id_y1_ | 0.00 | | |
| ICC | 0.61 | | |
| N _site_id_y1_ | 21 | | |
| N _rel_family_id_ | 2155 | | |
| Observations | 2491 | | |
| Marginal R^2^ / Conditional R^2^ | 0.046 / 0.632 | | |

Table S7 — Females: Effect of gonadal pubertal timing on youth depression controlling for adrenal pubertal timing. Base model with associated statistics. Note: IRR = incidence rate ratio. AIAN/NHPI = AIAN/NHPI = American Indian/Alaska Native/Native Hawaiian and other Pacific Islander.

| **Males: Effect of gonadal timing on youth depression, controlling for adrenal pubertal timing** | | | |
| --- | --- | --- | --- |
|  | **Base** | | |
| *Predictors* | *IRR* | *SE* | *P-Value* |
| (Intercept) | 0.737 | 0.027 | **<0.001** |
| Gonadal pubertal timing | 0.994 | 0.034 | 0.850 |
| Adrenal pubertal timing | 1.109 | 0.038 | **0.003** |
| Age | 1.042 | 0.031 | 0.161 |
| Race: Black | 0.684 | 0.083 | **0.002** |
| Race: Asian | 0.591 | 0.135 | **0.022** |
| Race: AIAN/NHPI | 0.959 | 0.425 | 0.925 |
| Race: Other | 1.113 | 0.189 | 0.528 |
| Race: Mixed | 1.165 | 0.118 | 0.132 |
| **Random Effects** | | | |
| σ^2^ | 0.87 | | |
| τ_00_ _rel_family_id_ | 1.28 | | |
| τ_00_ _site_id_y1_ | 0.00 | | |
| N _site_id_y1_ | 21 | | |
| N _rel_family_id_ | 2412 | | |
| Observations | 2752 | | |
| Marginal R^2^ / Conditional R^2^ | 0.033 / NA | | |

Table S8 — Males: Effect of gonadal pubertal timing on youth depression controlling for adrenal pubertal timing. Base model with associated statistics. Note: IRR = incidence rate ratio. AIAN/NHPI = AIAN/NHPI = American Indian/Alaska Native/Native Hawaiian and other Pacific Islander.

## Hypotheses 2 & 3: Whole brain exploratory analyses

We undertook exploratory analyses to identify any additional brain structural measures that may mediate the association between earlier pubertal timing and later increased depressive symptoms. Further, we also wanted to identify brain structural features related to pubertal timing and depressive symptoms in this large sample of early adolescents. We re-ran the base models specified in the pilot analyses (with the removal of WBV a covariate due to its potential effect on regional brain estimates (Mills et al., 2016)) using pubertal timing data from year 1, imaging data from year 2, and depressive symptom data from year 3. For this exploratory analysis, we used the base model set up (as per the approach taken in our pilot analyses), so that the models were consistent across the pilot and main analyses.

*Whole brain exploratory analyses: Brain structural associations with pubertal timing*

Standardised beta values for the pubertal timing — brain structure associations that remained significant after correction for multiple comparison (*p*_FDR_ ≤0.001) are reported in the main text (see Figure 6). Namely, significant associations were found for frontal (e.g., middle frontal gyri), temporal (e.g., the insula, bank of the superior temporal sulcus), and parietal (e.g., the precuneus, inferior and superior parietal gyri, paracentral gyrus) regions. Relevant statistics (ß values, standard errors, uncorrected and corrected p-values) for all pubertal timing – brain structure models are reported in Supplementary Data 9 (females) & 10 (males).

*Brain structural associations with depressive symptoms*

Beta values for the brain structure – depression symptoms associations that remained significant after correction for multiple comparison (*p*_FDR_ ≤0.05) are reported in the main text. We note that a less conservative threshold for multiple comparison correction was used here compared to the pubertal timing — brain structure models due to the more numerous and stronger associations found for the latter association. Relevant statistics for all brain structure — depression models can be found in Supplementary Data 11 (females) & 12 (males).

*Exploratory mediation analysis*

Unlike the pilot analyses, whereby we included any ROIs that were associated with increased depressive symptoms and/or earlier pubertal timing, we adopted a more streamlined approach in our exploratory analyses given that we had full access to the data and any findings would be reported as post-hoc. Therefore, we included any ROI that demonstrated a significant association (after correction for multiple comparisons, specified above) with *both* earlier pubertal timing at year 1 and increased depressive symptoms at year 3. The only brain measure that met this criterion was lower volume of the accumbens area in females. Model 1: Accumbens area ~ pubertal timing (Y ~ X): ß = -0.086, *p*_FDR_ = 0.001); Model 2: Depressive symptoms ~ accumbens area (Y ~ X): ß = -0.105, *p*_FDR_ = 0.02). In males, lower accumbens area volume was associated with increased depressive symptoms (ß = -0.104, *p*_FDR_= 0.013) but not with pubertal timing (ß = -0.02, *p*_FDR_= 0.36). However, for completeness, we tested whether the accumbens area mediated the association between earlier pubertal timing and later depression in both females and males. Results of this mediation analysis are illustrated in Figure 7 of the main text.

# Sensitivity Analyses

## Hypothesis 1: Multiple imputation of missing outcome and covariate data

For H1, multiple imputation by chained equations (MICE) was undertaken using the “mice” package in R (Buuren & Groothuis-Oudshoorn, 2011) to impute missing data for youth depression at year 3 (outcome), BMI at year 1 and parental mood at year 2. In our final sample (Females: N= 2533; Males: N = 2792) which included participants with complete puberty data and who had attended the year 3 follow-up appointment, there was no missing data for site, age, sex, or the population weighting score variables. We did not impute data for race/ethnicity so participants with missing data (N = 30 (females); N = 26 (males)) for this variable were not included in the imputation analysis. Further, we did not impute household income as this data was only collected at baseline and we were unable to find a suitable auxiliary variable (e.g., highest parental education) as participants with missing household income data were also missing parental education data.

Auxiliary variables were only included if they predicted the variable being imputed or missingness in this variable (to reduce the bias of variables being “missing not at random”) or if they had <40% missing data. The auxiliary variables included were: youth depression at baseline, year 1 and year 2 (measured via the CBCL withdrawn/depressed subscale); youth anxiety at baseline, year 1, year 2, and year 3; parent depression at baseline; and BMI at baseline. One hundred imputed datasets were created. Effect sizes from each imputed dataset were then pooled using Rubin’s rule. We note that missingness in our final sample was very low in both our base models (females = 42/2533 (1.66%); males = 40/2792 (1.34%)) and fully adjusted models (females = 107/2533 (4.22%); males = 89/2792 (3.19%).

Similar effect sizes were found when missing data was imputed and pooled for both females and males, as shown in Table S9 and S10 respectively.

| **Females: Pooled effect sizes for the association between pubertal timing on youth depression using MICE** | | | | | | |
| --- | --- | --- | --- | --- | --- | --- |
|  | **Base** | | | **Full** | | |
| *Predictors* | *Beta Estimate* | *SE* | *P-Value* | *Beta Estimate* | *SE* | *P-Value* |
| (Intercept) | -0.193 | 0.039 | **<0.001** | -0.448 | 0.258 | 0.083 |
| Pubertal timing | 0.273 | 0.031 | **<0.001** | 0.196 | 0.032 | **<0.001** |
| Age | 0.106 | 0.031 | **0.001** | 0.092 | 0.030 | **0.002** |
| Race: Black | -0.463 | 0.116 | **<0.001** | -0.343 | 0.118 | **0.004** |
| Race: Asian | -0.162 | 0.215 | 0.45 | 0.043 | 0.207 | 0.835 |
| Race: AIAN/NHPI | -0.046 | 0.375 | 0.902 | -0.188 | 0.367 | 0.609 |
| Race: Other | 0.213 | 0.162 | 0.189 | 0.298 | 0.160 | 0.062 |
| Race: Mixed | 0.186 | 0.096 | 0.052 | 0.109 | 0.093 | 0.241 |
| BMI |  |  |  | 0.101 | 0.032 | **0.001** |
| Household income: $5,000-$11,999 |  |  |  | 0.117 | 0.325 | 0.719 |
| Household income: $12,000-$15,999 |  |  |  | -0.108 | 0.327 | 0.742 |
| Household income: $16,000-$24,999 |  |  |  | 0.345 | 0.299 | 0.248 |
| Household income: $25,000-$34,999 |  |  |  | 0.172 | 0.286 | 0.548 |
| Household income: $35,000-$49,999 |  |  |  | 0.286 | 0.274 | 0.297 |
| Household income: $50,000-$74,999 |  |  |  | 0.257 | 0.268 | 0.337 |
| Household income: $75,000-$99,999 |  |  |  | 0.225 | 0.268 | 0.402 |
| Household income: $100,000-$199,999 |  |  |  | 0.290 | 0.262 | 0.269 |
| Household income: >$200,000 |  |  |  | 0.198 | 0.274 | 0.470 |
| Parent depressive symptoms |  |  |  | 0.408 | 0.030 | **<0.001** |

Table S9 — Females: Effect of pubertal timing on youth depression with imputed missing data. Base and fully adjusted models with associated statistics (pooled effect sizes using MICE). Note: AIAN/NHPI = AIAN/NHPI = American Indian/Alaska Native/Native Hawaiian and other Pacific Islander.

| **Males: Pooled effect sizes for the association between pubertal timing on youth depression using MICE** | | | | | | |
| --- | --- | --- | --- | --- | --- | --- |
|  | **Base** | | | **Full** | | |
| *Predictors* | *Beta Estimate* | *SE* | *P-Value* | *Beta Estimate* | *SE* | *P-Value* |
| (Intercept) | -0.302 | 0.037 | **<0.001** | -0.220 | 0.202 | 0.276 |
| Pubertal timing | 0.087 | 0.030 | **0.004** | 0.048 | 0.030 | 0.111 |
| Age | 0.041 | 0.029 | 0.166 | 0.022 | 0.028 | 0.430 |
| Race: Black | -0.378 | 0.121 | **0.002** | -0.377 | 0.121 | **0.002** |
| Race: Asian | -0.547 | 0.228 | 0.017 | -0.272 | 0.219 | 0.215 |
| Race: AIAN/NHPI | -0.104 | 0.437 | 0.812 | -0.007 | 0.420 | 0.987 |
| Race: Other | 0.073 | 0.169 | 0.667 | 0.048 | 0.164 | 0.771 |
| Race: Mixed | 0.151 | 0.101 | 0.134 | 0.074 | 0.096 | 0.443 |
| BMI |  |  |  | 0.119 | 0.029 | **<0.001** |
| Household income: $5,000-$11,999 |  |  |  | -0.441 | 0.287 | 0.125 |
| Household income: $12,000-$15,999 |  |  |  | -0.318 | 0.317 | 0.316 |
| Household income: $16,000-$24,999 |  |  |  | 0.140 | 0.240 | 0.559 |
| Household income: $25,000-$34,999 |  |  |  | 0.003 | 0.234 | 0.989 |
| Household income: $35,000-$49,999 |  |  |  | 0.072 | 0.220 | 0.744 |
| Household income: $50,000-$74,999 |  |  |  | -0.084 | 0.213 | 0.693 |
| Household income: $75,000-$99,999 |  |  |  | -0.100 | 0.213 | 0.639 |
| Household income: $100,000-$199,999 |  |  |  | -0.118 | 0.208 | 0.571 |
| Household income: >$200,000 |  |  |  | -0.094 | 0.220 | 0.668 |
| Parent depressive symptoms |  |  |  | 0.442 | 0.028 | **<0.001** |

Table S10 — Males: Effect of pubertal timing on youth depression with imputed missing data. Base and fully adjusted models with associated statistics (pooled effect sizes using MICE). Note: AIAN/NHPI = AIAN/NHPI = American Indian/Alaska Native/Native Hawaiian and other Pacific Islander.

## Hypothesis 1: Controlling for earlier youth depression

We examined the association between earlier pubertal timing and the potential change (or rather worsening) of depressive symptoms between timepoints (i.e., Year 1 and Year 3) by including Year 1 youth depressive symptoms as an additional covariate in our base and fully adjusted models.

For females, we found earlier pubertal timing was significantly associated with worsening depressive symptoms over time (Base model: ß = 0.17 [IRR = 1.17]; *p* <0.001; fully adjusted model: ß = 0.16 [IRR = 1.73]; *p* <0.001). For males, earlier pubertal timing was not significantly associated with worsening depressive symptoms over time (Base model: ß = 0.05 [IRR = 1.05]; *p* =0.06; fully adjusted model: ß = 0.05 [IRR = 1.05]; *p* = 0.06). Base and fully adjusted models are reported below in Table X (females) and X (males).

| **Females: Effect of pubertal timing on youth depression controlling for earlier youth depression** | | | | | | |
| --- | --- | --- | --- | --- | --- | --- |
|  | **Base** | | | **Full** | | |
| *Predictors* | *IRR* | *SE* | *P-Value* | *IRR* | *SE* | *P-Value* |
| (Intercept) | 1.000 | 0.034 | 1.000 | 0.635 | 0.152 | 0.058 |
| Pubertal timing | 1.173 | 0.032 | **<0.001** | 1.173 | 0.034 | **<0.001** |
| Age | 1.064 | 0.029 | **0.021** | 1.076 | 0.030 | **0.009** |
| Race: Black | 0.761 | 0.076 | **0.006** | 0.809 | 0.087 | **0.050** |
| Race: Asian | 0.950 | 0.178 | 0.783 | 1.015 | 0.200 | 0.938 |
| Race: AIAN/NHPI | 0.971 | 0.313 | 0.928 | 1.000 | 0.336 | 0.999 |
| Race: Other | 1.047 | 0.149 | 0.745 | 1.153 | 0.172 | 0.340 |
| Race: Mixed | 1.089 | 0.090 | 0.300 | 1.061 | 0.090 | 0.487 |
| Youth depressive symptoms(Y 1) | 1.613 | 0.036 | **<0.001** | 1.581 | 0.038 | **<0.001** |
| Household income: $5,000-$11,999 |  |  |  | 1.479 | 0.440 | 0.187 |
| Household income: $12,000-$15,999 |  |  |  | 1.139 | 0.349 | 0.671 |
| Household income: $16,000-$24,999 |  |  |  | 1.501 | 0.415 | 0.141 |
| Household income: $25,000-$34,999 |  |  |  | 1.035 | 0.275 | 0.898 |
| Household income: $35,000-$49,999 |  |  |  | 1.382 | 0.351 | 0.202 |
| Household income: $50,000-$74,999 |  |  |  | 1.328 | 0.330 | 0.252 |
| Household income: $75,000-$99,999 |  |  |  | 1.257 | 0.312 | 0.357 |
| Household income: $100,000-$199,999 |  |  |  | 1.367 | 0.332 | 0.199 |
| Household income: >$200,000 |  |  |  | 1.366 | 0.346 | 0.219 |
| Parent depressive symptoms |  |  |  | 1.287 | 0.037 | **<0.001** |
| **Random Effects** | | | | | | |
| σ^2^ | 0.81 | | | 0.81 | | |
| τ_00_ | 0.80 _rel_family_id_ | | | 0.80 _rel_family_id_ | | |
|  | 0.00 _site_id_y1_ | | | 0.00 _site_id_y1_ | | |
| N | 21 _site_id_y1_ | | | 21 _site_id_y1_ | | |
|  | 2155 _rel_family_id_ | | | 2137 _rel_family_id_ | | |
| Observations | 2491 | | | 2469 | | |
| Marginal R^2^ / Conditional R^2^ | 0.264 / NA | | | 0.337 / NA | | |

Table S11— Females: Effect of pubertal timing on youth depression controlling for earlier youth depression (at Year 1). Note: IRR = incidence rate ratio. AIAN/NHPI = AIAN/NHPI = American Indian/Alaska Native/Native Hawaiian and other Pacific Islander.

| **Males: Effect of pubertal timing on youth depression controlling for earlier youth depression** | | | | | | |
| --- | --- | --- | --- | --- | --- | --- |
|  | **Base** | | | **Full** | | |
| *Predictors* | *IRR* | *SE* | *P-Value* | *IRR* | *SE* | *P-Value* |
| (Intercept) | 0.527 | 0.020 | **<0.001** | 0.531 | 0.096 | **<0.001** |
| Pubertal timing | 1.052 | 0.028 | 0.057 | 1.053 | 0.028 | 0.056 |
| Age | 1.003 | 0.026 | 0.902 | 0.998 | 0.026 | 0.944 |
| Race: Black | 0.751 | 0.078 | **0.006** | 0.797 | 0.087 | **0.037** |
| Race: Asian | 0.750 | 0.149 | 0.147 | 0.830 | 0.168 | 0.357 |
| Race: AIAN/NHPI | 1.039 | 0.382 | 0.916 | 1.179 | 0.434 | 0.655 |
| Race: Other | 1.018 | 0.148 | 0.905 | 1.060 | 0.156 | 0.692 |
| Race: Mixed | 1.131 | 0.097 | 0.152 | 1.098 | 0.094 | 0.274 |
| Youth depressive symptoms (Y 1) | 1.394 | 0.016 | **<0.001** | 1.348 | 0.017 | **<0.001** |
| Household income: $5,000-$11,999 |  |  |  | 0.815 | 0.207 | 0.421 |
| Household income: $12,000-$15,999 |  |  |  | 0.732 | 0.208 | 0.273 |
| Household income: $16,000-$24,999 |  |  |  | 1.017 | 0.217 | 0.936 |
| Household income: $25,000-$34,999 |  |  |  | 1.014 | 0.211 | 0.945 |
| Household income: $35,000-$49,999 |  |  |  | 1.176 | 0.229 | 0.407 |
| Household income: $50,000-$74,999 |  |  |  | 0.962 | 0.181 | 0.836 |
| Household income: $75,000-$99,999 |  |  |  | 0.994 | 0.188 | 0.976 |
| Household income: $100,000-$199,999 |  |  |  | 1.039 | 0.191 | 0.836 |
| Household income: >$200,000 |  |  |  | 1.025 | 0.200 | 0.901 |
| Parent depressive symptoms |  |  |  | 1.273 | 0.033 | **<0.001** |
| **Random Effects** | | | | | | |
| σ^2^ | 0.87 | | | 0.87 | | |
| τ_00_ | 0.70 _rel_family_id_ | | | 0.65 _rel_family_id_ | | |
|  | 0.00 _site_id_y1_ | | | 0.00 _site_id_y1_ | | |
| N | 21 _site_id_y1_ | | | 21 _site_id_y1_ | | |
|  | 2412 _rel_family_id_ | | | 2393 _rel_family_id_ | | |
| Observations | 2752 | | | 2733 | | |
| Marginal R^2^ / Conditional R^2^ | 0.291 / NA | | | 0.331 / NA | | |

Table S12— Males: Effect of pubertal timing on youth depression controlling for earlier youth depression (at Year 1). Note: IRR = incidence rate ratio. AIAN/NHPI = AIAN/NHPI = American Indian/Alaska Native/Native Hawaiian and other Pacific Islander.

## Hypothesis 1: Population weight raked propensity score

For H1, we also compared models with and without a population weighting score included as a weight in our generalised linear mixed model in Table S13 (females) and Table S14 (males). The inclusion of a population weighting score seemed to inflate the effect sizes and standard errors of minority race/ethnicity groups (e.g., AIAN/NHPI), which may be due to the small number of individuals (0.85% (females)/0.57% (males)) who reported this as their race/ethnicity in ABCD. Given that our main research questions did not examine race interaction effects, we decided to remove the population weighting score from our main analyses and report it as sensitivity analyses instead.

| **Females: Effect of pubertal timing on youth depression** | | | | | | | | | |
| --- | --- | --- | --- | --- | --- | --- | --- | --- | --- |
|  | **Base** | | | **Full** | | | **Full (weighted)** | | |
| *Predictors* | *IRR* | *SE* | *P-Value* | *IRR* | *SE* | *P-Value* | *IRR* | *SE* | *P-Value* |
| (Intercept) | 0.821 | 0.032 | **<0.001** | 0.636 | 0.172 | 0.093 | 0.002 | 0.002 | **<0.001** |
| Pubertal timing | 1.313 | 0.041 | **<0.001** | 1.220 | 0.040 | **<0.001** | 1.217 | 0.004 | **<0.001** |
| Age | 1.111 | 0.034 | **0.001** | 1.097 | 0.034 | **0.002** | 1.222 | 0.004 | **<0.001** |
| Race: Black | 0.630 | 0.073 | **<0.001** | 0.723 | 0.088 | **0.007** | 0.257 | 0.138 | **0.011** |
| Race: Asian | 0.854 | 0.187 | 0.471 | 1.032 | 0.222 | 0.884 | 0.942 | 0.914 | 0.951 |
| Race: AIAN/NHPI | 0.961 | 0.360 | 0.915 | 0.884 | 0.328 | 0.739 | 299.294 | 175.494 | **<0.001** |
| Race: Other | 1.299 | 0.213 | 0.110 | 1.497 | 0.246 | **0.014** | 3.100 | 0.121 | **<0.001** |
| Race: Mixed | 1.217 | 0.116 | **0.040** | 1.138 | 0.107 | 0.169 | 1.598 | 0.024 | **<0.001** |
| BMI |  |  |  | 1.105 | 0.036 | **0.002** | 1.238 | 0.004 | **<0.001** |
| Household income: $5,000-$11,999 |  |  |  | 1.094 | 0.371 | 0.792 | 6.451 | 9.889 | 0.224 |
| Household income: $12,000-$15,999 |  |  |  | 0.850 | 0.297 | 0.642 | 4.560 | 5.604 | 0.217 |
| Household income: $16,000-$24,999 |  |  |  | 1.379 | 0.430 | 0.303 | 4.523 | 5.558 | 0.219 |
| Household income: $25,000-$34,999 |  |  |  | 1.107 | 0.331 | 0.735 | 0.069 | 0.087 | **0.033** |
| Household income: $35,000-$49,999 |  |  |  | 1.339 | 0.383 | 0.307 | 8.615 | 10.584 | 0.080 |
| Household income: $50,000-$74,999 |  |  |  | 1.300 | 0.364 | 0.349 | 36.266 | 44.236 | **0.003** |
| Household income: $75,000-$99,999 |  |  |  | 1.245 | 0.349 | 0.433 | 0.408 | 0.498 | 0.462 |
| Household income: $100,000-$199,999 |  |  |  | 1.338 | 0.367 | 0.288 | 3.847 | 4.667 | 0.267 |
| Household income: >$200,000 |  |  |  | 1.255 | 0.358 | 0.427 | 1.976 | 2.491 | 0.589 |
| Parent depressive symptoms |  |  |  | 1.500 | 0.046 | **<0.001** | 1.144 | 0.016 | **<0.001** |
| **Random Effects** | | | | | | | | | |
| σ^2^ | 0.81 | | | 0.81 | | | 4.76 | | |
| τ_00_ | 1.29 _rel_family_id_ | | | 1.12 _rel_family_id_ | | | 43.19 _rel_family_id_ | | |
|  | 0.00 _site_id_y1_ | | | 0.00 _site_id_y1_ | | | 0.00 _site_id_y1_ | | |
| ICC |  | | |  | | | 0.90 | | |
| N | 21 _site_id_y1_ | | | 21 _site_id_y1_ | | | 21 _site_id_y1_ | | |
|  | 2155 _rel_family_id_ | | | 2105 _rel_family_id_ | | | 2105 _rel_family_id_ | | |
| Observations | 2491 | | | 2426 | | | 2426 | | |
| Marginal R^2^ / Conditional R^2^ | 0.111 / NA | | | 0.252 / NA | | | 0.063 / 0.907 | | |

Table S13 — Females: Base model, fully adjusted model and fully adjusted model with population propensity score weight included as a weight in the model with associated statistics. Note: IRR = incidence rate ratio. AIAN/NHPI = AIAN/NHPI = American Indian/Alaska Native/Native Hawaiian and other Pacific Islander.

| **Males: Effect of pubertal timing on youth depression** | | | | | | | | | |
| --- | --- | --- | --- | --- | --- | --- | --- | --- | --- |
|  | **Base** | | | **Full** | | | **Full (weighted)** | | |
| *Predictors* | *IRR* | *SE* | *P-Value* | *IRR* | *SE* | *P-Value* | *IRR* | *SE* | *P-Value* |
| (Intercept) | 0.738 | 0.027 | **<0.001** | 0.864 | 0.178 | 0.478 | 0.003 | 0.003 | **<0.001** |
| Pubertal timing | 1.088 | 0.033 | **0.006** | 1.045 | 0.032 | 0.151 | 1.011 | 0.003 | **<0.001** |
| Age | 1.041 | 0.031 | 0.167 | 1.025 | 0.029 | 0.397 | 1.061 | 0.003 | **<0.001** |
| Race: Black | 0.685 | 0.083 | **0.002** | 0.671 | 0.083 | **0.001** | 0.518 | 0.288 | 0.237 |
| Race: Asian | 0.586 | 0.134 | **0.019** | 0.735 | 0.167 | 0.175 | 0.012 | 0.012 | **<0.001** |
| Race: AIAN/NHPI | 0.950 | 0.419 | 0.908 | 1.046 | 0.443 | 0.915 | 0.012 | 0.030 | 0.074 |
| Race: Other | 1.096 | 0.186 | 0.590 | 1.036 | 0.173 | 0.834 | 1.599 | 0.074 | **<0.001** |
| Race: Mixed | 1.165 | 0.118 | 0.130 | 1.087 | 0.106 | 0.391 | 87.971 | 19.418 | **<0.001** |
| BMI |  |  |  | 1.126 | 0.033 | **<0.001** | 1.245 | 0.004 | **<0.001** |
| Household income: $5,000-$11,999 |  |  |  | 0.677 | 0.198 | 0.182 | 0.055 | 0.077 | **0.040** |
| Household income: $12,000-$15,999 |  |  |  | 0.722 | 0.232 | 0.311 | 0.121 | 0.188 | 0.173 |
| Household income: $16,000-$24,999 |  |  |  | 1.051 | 0.257 | 0.838 | 1.760 | 1.816 | 0.584 |
| Household income: $25,000-$34,999 |  |  |  | 0.909 | 0.218 | 0.692 | 1.695 | 1.748 | 0.609 |
| Household income: $35,000-$49,999 |  |  |  | 0.989 | 0.222 | 0.962 | 2.744 | 2.830 | 0.328 |
| Household income: $50,000-$74,999 |  |  |  | 0.857 | 0.186 | 0.476 | 0.101 | 0.104 | **0.026** |
| Household income: $75,000-$99,999 |  |  |  | 0.839 | 0.182 | 0.419 | 6.749 | 6.927 | 0.063 |
| Household income: $100,000-$199,999 |  |  |  | 0.819 | 0.174 | 0.347 | 0.624 | 0.640 | 0.645 |
| Household income: >$200,000 |  |  |  | 0.840 | 0.189 | 0.436 | 0.970 | 1.047 | 0.978 |
| Parent depressive symptoms |  |  |  | 1.562 | 0.045 | **<0.001** | 1.302 | 0.014 | **<0.001** |
| **Random Effects** | | | | | | | | | |
| σ^2^ | 0.87 | | | 0.87 | | | 5.53 | | |
| τ_00_ | 1.28 _rel_family_id_ | | | 1.04 _rel_family_id_ | | | 43.82 _rel_family_id_ | | |
|  | 0.00 _site_id_y1_ | | | 0.00 _site_id_y1_ | | | 0.28 _site_id_y1_ | | |
| ICC |  | | |  | | | 0.89 | | |
| N | 21 _site_id_y1_ | | | 21 _site_id_y1_ | | | 21 _site_id_y1_ | | |
|  | 2412 _rel_family_id_ | | | 2369 _rel_family_id_ | | | 2369 _rel_family_id_ | | |
| Observations | 2752 | | | 2703 | | | 2703 | | |
| Marginal R^2^ / Conditional R^2^ | 0.030 / NA | | | 0.224 / NA | | | 0.089 / 0.899 | | |

Table S14 — Males: Base model, fully adjusted model and fully adjusted model with population propensity score weight included as a weight in the model with associated statistics. Note: IRR = incidence rate ratio. AIAN/NHPI = AIAN/NHPI = American Indian/Alaska Native/Native Hawaiian and other Pacific Islander.

## Pilot analyses

### Statistical model specifications

Given inconsistent findings in terms of the brain regions associated with pubertal development and depressive symptoms in adolescence, we conducted pilot analysis on baseline data from the ABCD Study to identify ROIs for our second and third hypotheses. We note that although our main analyses used imaging data from Year 2 and depressive symptoms from the Year 3 follow up, the pilot analyses used baseline data *only* for all measures (N = 9,339, males = 4802, females = 4537, mean age = 9.91 years, SD = 0.62). This was to avoid handling any of the follow-up data given that the study is a registered report. Due to the non-longitudinal nature of the pilot analyses, we used complete case analysis.

The pilot analyses consisted of identifying ROIs that were significantly associated with *baseline* measures of 1) pubertal timing and/or 2) depressive symptoms. An association was considered significant if the nominal (un-corrected) p-value ≤0.0001 for the pubertal timing-brain models and a p-value ≤0.005 for the depression-brain models. Nominal thresholds were chosen since these were pilot analyses conducted to inform the main analyses. We note that due to the more numerous and stronger associations observed for the pubertal timing-brain models, a more conservative p-value threshold was chosen to limit the number of ROIs carried through to the main analyses.

Associations between pubertal timing (indexed via PDS) and brain structural measures (cortical, subcortical, and white matter microstructure) were examined using GLMs via the ‘lmerTest’ (Kuznetsova et al., 2017). Covariates included: age, race/ethnicity, WBV, DTI motion, (for DTI models) as fixed effects and individual ID and scanner ID as random effects.

Associations between depressive symptoms and brain structural measures were examined using the same model specifications as described above.

The analyses of associations with brain structural measures followed a hierarchical order from global measures at the whole-brain level to individual structures. For cortical measures, this included whole brain cortical volume, mean thickness, total surface area, mean sulcal depth, followed by individual brain regions. For subcortical measures, this included volumes for subcortical regions as specified in the FreeSurfer segmentation. For white matter microstructural measures (FA/MD), the global ‘g’ measures were first tested, followed by individual tracts.

These brain measures were examined for 1) pubertal timing and 2) depressive symptoms. Models were run separately for males and females. See Table S15 for model specifications.

| **Type** | **Measures** | **Number of variables** | **Covariates (Base model)** |
| --- | --- | --- | --- |
| Global  brain measures | Mean whole-brain cortical thickness | 1 unilateral | Fixed: Age + race/ethnicity.  Random: Family ID +scanner ID |
|  | Total whole-brain surface area | 1 unilateral |  |
|  | Mean whole-brain sulcal depth | 1 unilateral |  |
|  | Total whole-brain volume | 1 unilateral |  |
|  | Global total white matter fractional anisotropy | 1 unilateral |  |
|  | Global total white matter mean diffusivity | 1 unilateral |  |
| Regional brain measures | Cortical thickness | 34 bilateral | Fixed: Age + race/ethnicity + WBV.  Random: Family ID +scanner ID |
|  | Cortical surface area | 34 bilateral |  |
|  | Cortical sulcal depth | 34 bilateral |  |
|  | Cortical volume  Subcortical regions | 34 bilateral  16 bilateral |  |
|  | White matter fractional anisotropy | 14 bilateral, 3 unilateral | Fixed: Age + race/ethnicity + DTI motion.  Random: Family ID +scanner ID |
|  | White matter mean diffusivity | 14 bilateral, 3 unilateral |  |

Table S15 — Pilot analyses: Model specifications for brain structural measures.

To examine pubertal timing ~ brain structure associations, we ran **6** models for global brain measures, and **184** models for regional brain measures for males and females separately.

To test associations between depressive symptoms and brain structure, we ran **6** models for global brain measures and **184** models for regional brain measures for both males and females separately.

### Pilot results

**The association between pubertal timing and brain structural measures at baseline**

*Global brain metrics*

Earlier pubertal timing was associated with lower global cortical thickness and sulcal depth in females (β range: -0⋅054 to -0.066; p_uncorrected_: = 5.55x10^-6^ to 5.12x10^-5^). No significant relationships were found in males.

*Regional brain metrics*

For females, earlier pubertal timing was associated with decreased cortical thickness and volume in temporal and frontal regions, and increased cortical volume in the ventral diencephalon (β range: -0.0425 to 0.0931; p_uncorrected_ range: 6.73x10^-7^  to 0.0001) (see Table S16 and Figure S1).

For males, earlier pubertal timing was also associated with decreased cortical thickness in the lateral orbitofrontal cortex (β: -0.0525; p_uncorrected_ : 0.0001) (see Table S16).

Regions meeting criteria for ROIs (p≤0.0001) are reported in Table S16 and illustrated in Figure S1. See Supplementary Data 1&2 for full details of all models for males and females, respectively.

Figure S1 — Pilot results: Cortical and subcortical regions of interest for the pubertal timing ~ brain structure association.


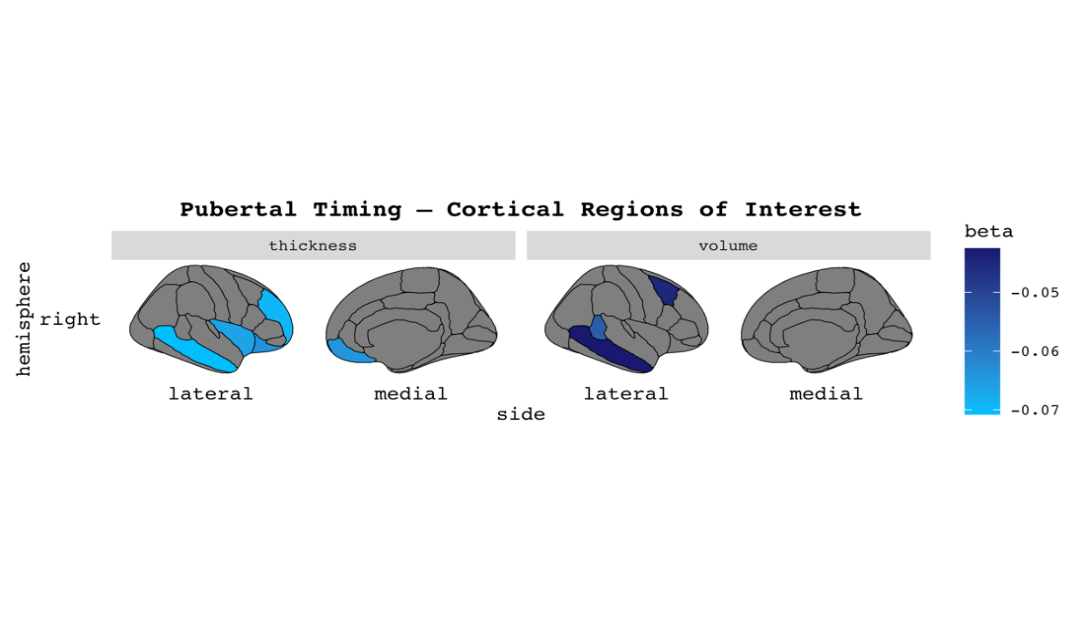

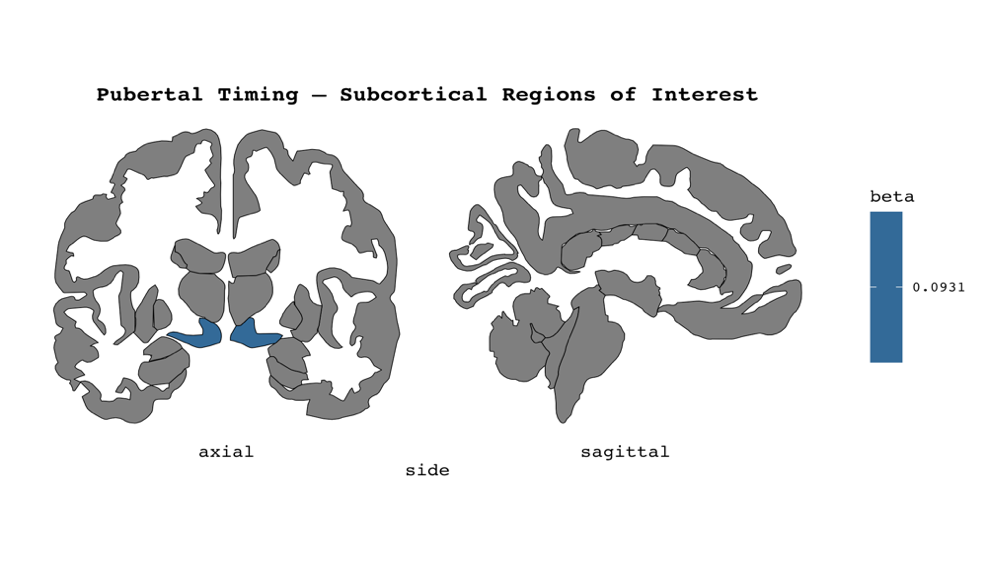


| **Pubertal timing** | **Brain Structure** | **beta** | **std** | **t.value** | **p_uncorrected_** |
| --- | --- | --- | --- | --- | --- |
| **Females** |  |  |  |  |  |
| PDS | Global cortical thickness | -0.0659 | 0.0145 | -4.5476 | 5.55x10^-6^ |
|  | Global sulcal depth | -0.0540 | 0.0133 | -4.0536 | 5.12x10^-5^ |
|  | Insula (thickness) | -0.0663 | 0.0148 | -4.4816 | 7.58x10^-6^ |
|  | Lateral orbitofrontal cortex (thickness) | -0.0635 | 0.0150 | -4.2293 | 2.39x10^-5^ |
|  | Middle temporal gyrus (thickness) | -0.0708 | 0.0142 | -4.9754 | 6.73x10^-7^ |
|  | Medial orbitofrontal cortex (thickness) | -0.0640 | 0.0148 | -4.3262 | 1.55x10^-5^ |
|  | Rostral middle frontal gyrus (thickness) | -0.0696 | 0.0149 | -4.6687 | 3.11x10^-6^ |
|  | Bank of the superior temporal sulcus (volume) | -0.0545 | 0.0129 | -4.2148 | 2.54x10^-5^ |
|  | Caudal middle frontal gyrus (volume) | -0.0449 | 0.0120 | -3.7279 | 0.0001 |
|  | middle temporal gyrus (volume) | -0.0425 | 0.0105 | -4.0528 | 5.14x10^-5^ |
|  | Ventral diencephalon (volume) | 0.0931 | 0.0101 | 9.2557 | 3.12x10^-20^ |
| **Males** |  |  |  |  |  |
| PDS | Lateral orbitofrontal cortex (thickness) | -0.0525 | 0.0141 | -3.7237 | 0.0001 |

Table S16 — Pilot results: Pubertal timing-brain structure models and associated statistics with significant ROI associations.

**The association between depressive symptoms and brain structural measures at baseline**

*Global brain metrics*

Increased depressive symptoms were associated with reduced global cortical volume and surface area for males and females and reduced global FA for females only (β range: -0.0636 to -0.0290; p_uncorrected_ range: 5.67x10^-8^ to 0.0037). No significant relationships were observed in males.

*Regional brain metrics*

For females, increased depressive symptoms were associated with lower FA in the corpus callosum and parietal superior cortico-striate tract, and increased sulcal depth in the pars orbitalis (β range: -0.0280 to 0.0375; p_uncorrected_ range: 0.0051 to 0.0057).

For males, increased depressive symptoms were associated with decreased cortical surface area and volume in the postcentral gyrus, and reduced volume in the middle temporal gyrus (β range: -0.0256 to -0.0285; p_uncorrected_ range: 0.0014 to 0.0042).

Regions that met criteria to be classified as ROIs (uncorrected p-value ≤0.005) are shown in Table S17. Cortical ROIs are also illustrated in Figure S2. Please see Supplementary Data 3&4 for a complete list of all depression-brain structure models and associated statistics.


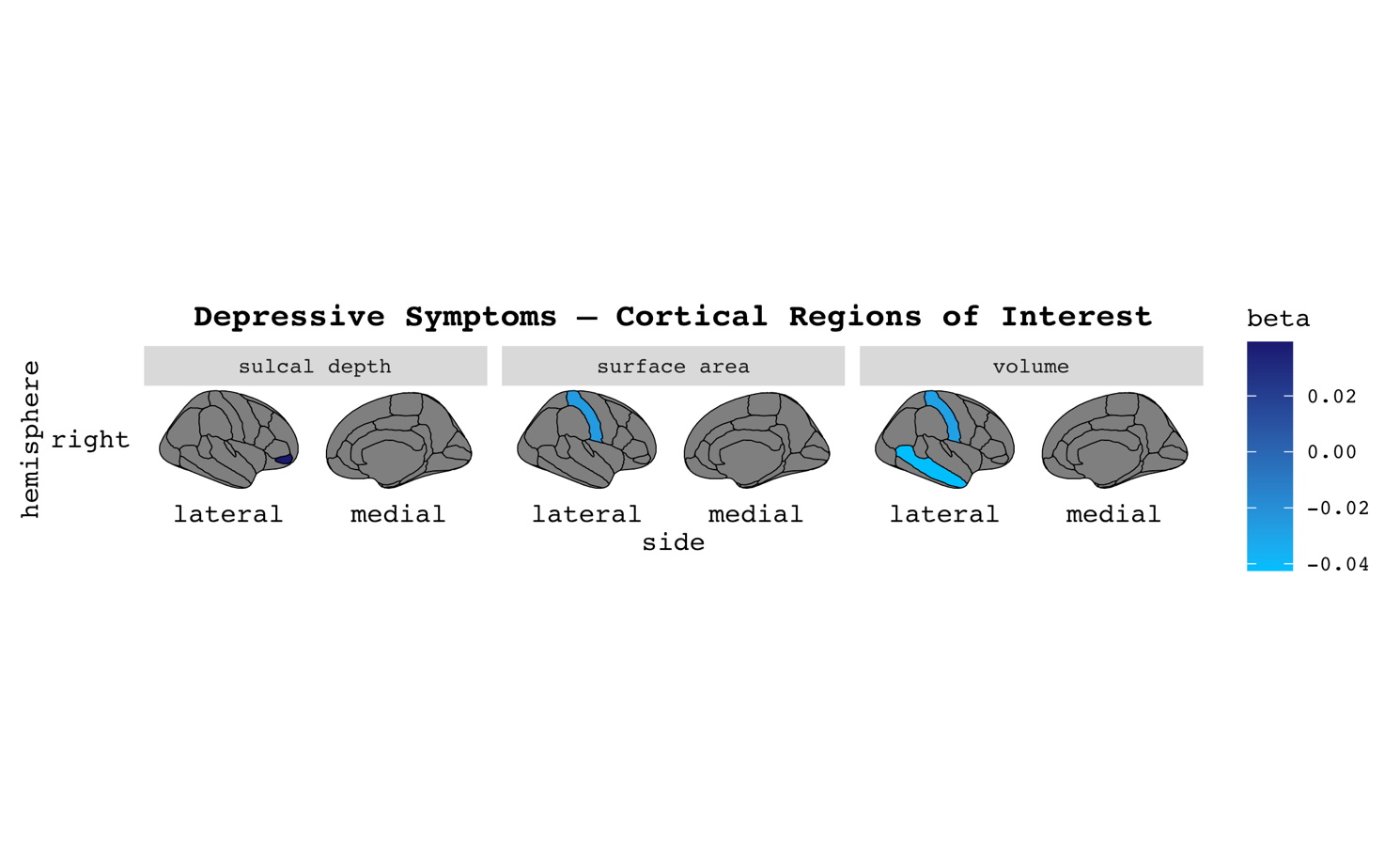


Figure S2 — Pilot results: Cortical and subcortical regions of interest for the depressive symptoms ~ brain structure association.

Note: DTI ROIs (corpus callosum and parietal superior corticostriate tract) are not illustrated in Figure S2 but listed in Table S17.

| **Depressive**  **Symptoms** | **Brain Structure** | **beta** | **std** | **t.value** | **p_uncorrected_** |
| --- | --- | --- | --- | --- | --- |
| **Females** |  |  |  |  |  |
| CBCL Withdrawn Depressed (raw score) | Global surface area | -0.0396 | 0.0122 | -3.2330 | 0.0012 |
|  | Global cortical volume | -0.0393 | 0.0122 | -3.2238 | 0.0013 |
|  | Global FA | -0.0290 | 0.0100 | -2.9071 | 0.0037 |
|  | Pars orbitalis  (Sulcal depth) | 0.0393 | 0.0133 | 2.9420 | 0.0033 |
|  | Corpus Callosum (FA) | -0.0280 | 0.0101 | -2.7656 | 0.0057 |
|  | Parietal superior corticostriate (FA) | -0.0327 | 0.0117 | -2.7990 | 0.0051 |
| **Males** |  |  |  |  |  |
| CBCL Withdrawn Depressed (raw score) | Global cortical surface area | -0.0607 | 0.0118 | -5.1263 | 3.06x10^-7^ |
|  | Global cortical volume | -0.0636 | 0.0117 | -5.4364 | 5.67x10^-8^ |
|  | Postcentral gyrus (surface area) | -0.0256 | 0.0090 | -2.8623 | 0.0042 |
|  | Middle temporal gyrus (volume) | -0.0283 | 0.0089 | -3.1887 | 0.0014 |
|  | Postcentral gyrus (volume) | -0.0285 | 0.0093 | -3.0636 | 0.0022 |

Table S17 — Pilot results: Depressive symptoms ~ brain structure models and associated statistics with significant ROI associations.

## Data access

N.M and X.S have had access to the ABCD annual curated data release 2.0.1 through their project entitled, “Brain structural associations with depression in a large early adolescent sample (the ABCD Study) (Shen et al., 2021). N.M and X.S also have access to the curated data releases 3.0 and 4.0. X.S looked at the baseline depression measures outlined in the current project. N.M has accessed the **baseline** puberty, depression, imaging, and socio-environmental variables outlined in the current project for the purposes of data quality control and pre-processing. N.M looked at year 1, year 2 and year 3 follow up data to determine sample sizes for the main analyses. N.M and all co-authors self-certify that they did not observe any of the statistical models outlined in the confirmatory analysis until after the in-principle acceptance was issued.

## References

Buuren, S. van, & Groothuis-Oudshoorn, K. (2011). mice: Multivariate Imputation by Chained Equations in R. *Journal of Statistical Software*, *45*, 1–67. https://doi.org/10.18637/jss.v045.i03

Herting, M. M., Uban, K. A., Robledo Gonzalez, M., Baker, F. C., Kan, E. C., Thompson, W. K., Granger, D. A., Albaugh, M. D., Anokhin, A. P., Bagot, K. S., Banich, M. T., Barch, D. M., Baskin-Sommers, A., Breslin, F. J., Casey, B. J., Chaarani, B., Chang, L., Clark, D. B., Cloak, C. C., … Edu, K. (2021). Correspondence Between Perceived Pubertal Development and Hormone Levels in 9-10 Year-Olds From the Adolescent Brain Cognitive Development Study. *Article*, *11*, 1–1. https://doi.org/10.3389/fendo.2020.549928

Kuznetsova, A., Brockhoff, P. B., & Christensen, R. H. B. (2017). lmerTest Package: Tests in Linear Mixed Effects Models. *Journal of Statistical Software*, *82*, 1–26. https://doi.org/10.18637/jss.v082.i13

Mills, K. L., Goddings, A. L., Herting, M. M., Meuwese, R., Blakemore, S. J., Crone, E. A., Dahl, R. E., Güroğlu, B., Raznahan, A., Sowell, E. R., & Tamnes, C. K. (2016). Structural brain development between childhood and adulthood: Convergence across four longitudinal samples. *NeuroImage*, *141*, 273–281. https://doi.org/10.1016/j.neuroimage.2016.07.044

Shirtcliff, E. A., Dahl, R. E., & Pollak, S. D. (2009). Pubertal development: Correspondence between hormonal and physical development. *Child Development*, *80*(2), 327–337. https://doi.org/10.1111/j.1467-8624.2009.01263.x
